# Anionic nanoplastic contaminants promote Parkinson’s disease–associated α-synuclein aggregation

**DOI:** 10.1126/sciadv.adi8716

**Published:** 2023-11-17

**Authors:** Zhiyong Liu, Arpine Sokratian, Addison M. Duda, Enquan Xu, Christina Stanhope, Amber Fu, Samuel Strader, Huizhong Li, Yuan Yuan, Benjamin G. Bobay, Joana Sipe, Ketty Bai, Iben Lundgaard, Na Liu, Belinda Hernandez, Catherine Bowes Rickman, Sara E. Miller, Andrew B. West

**Affiliations:** ^1^ Duke Center for Neurodegeneration and Neurotheraputics, Duke University, Durham, NC, USA.; ^2^ Department of Chemistry, Duke University, Durham, NC, USA.; ^3^ Department of Civil and Environmental Engineering, Duke University, Durham, NC, USA.; ^4^ Department of Experimental Medical Science, Lund University, Lund, Sweden.; ^5^ Wallenberg Center for Molecular Medicine, Lund University, Lund, Sweden.; ^6^ Department of Ophthalmology and Cell Biology, Duke University, Durham, NC, USA.; ^7^ Department of Pathology, Duke University, Durham, NC, USA.; ^8^ Aligning Science Across Parkinson’s (ASAP) Collaborative Research Network, Chevy Chase, MD, USA.

## Abstract

Recent studies have identified increasing levels of nanoplastic pollution in the environment. Here, we find that anionic nanoplastic contaminants potently precipitate the formation and propagation of α-synuclein protein fibrils through a high-affinity interaction with the amphipathic and non-amyloid component (NAC) domains in α-synuclein. Nanoplastics can internalize in neurons through clathrin-dependent endocytosis, causing a mild lysosomal impairment that slows the degradation of aggregated α-synuclein. In mice, nanoplastics combine with α-synuclein fibrils to exacerbate the spread of α-synuclein pathology across interconnected vulnerable brain regions, including the strong induction of α-synuclein inclusions in dopaminergic neurons in the substantia nigra. These results highlight a potential link for further exploration between nanoplastic pollution and α-synuclein aggregation associated with Parkinson’s disease and related dementias.

## Teaser

Small plastic particles stimulate Parkinson’s disease-associated features in the brain.

## INTRODUCTION

Increased plastic production and deposition of waste worldwide have resulted in microplastic contaminants in water and food supplies (*1*–*3*). Microplastics, defined as particles smaller than 5 mm in diameter, can also include smaller nanoplastics (less than 1 μm). Single-use polystyrene products, such as foam packing materials, cups, and cutlery, are largely responsible for widespread polystyrene waste that contributes to overall plastic pollution (*4*, *5*). Negatively charged small plastic particles are found in marine environments as a presumed consequence of ultraviolet radiation exposures and plastic erosion (*6*).

In experimental studies, anionic polystyrene nanoplastics induce endothelial leakiness and potentially compromise the blood-brain barrier in mammals by intercalating with cadherin dimers (*7*). A recent study found that polystyrene plastic pollution, among a few other types of plastic, circulates in the blood of most adults tested (*8*). In that study, a pyrolysis mass spectrometry approach suggested that the concentration of polystyrene ranged from ~1 to over 4 μg/ml in about a quarter of blood samples analyzed from healthy controls, although particle sizes of nanoplastics in the brain have not been determined. In models, polystyrene nanoplastics have been reported to accumulate in the brain and penetrate into the parenchyma (*9*–*13*). Acrylic latex microspheres (0.02 to 0.2 μm in diameter), administered through intracranial injections, have been widely used as neural-retrograde trafficking tracers (*14*, *15*). It is unclear whether other chemical forms of nanoplastics, including charged polystyrene, can be internalized and trafficked in neurons once these contaminants are inside the brain (*13*, *16*). Nonetheless, nanoparticles could hijack parts of the endocytic trafficking pathway to potentially travel along different circuits in the brain (*17*).

Among the fastest growing neurological disorders in the world, Parkinson’s disease (PD) and related dementias are pathologically defined by the accumulation of α-synuclein protein in vulnerable neurons in the brain (*18*). Increased longevity, heritable factors, and prolonged exposure to environmental challenges of largely unknown origins are suspected as principal driving factors for disease risk and progression (*19*). α-Synuclein is typically a low-molecular-weight protein, but in PD-affected tissues, electron microscopy studies show that fibril structures interact with damaged lipids from various organelles, such as lysosomes and mitochondria (*20*). In vitro studies show that some types of charged nanoparticles can inhibit α-synuclein fibril nucleation and fibril-seeded elongation (*21*). Graphene nanoparticles (less than 100 nm in diameter) administered to neuronal cultures and mice in preformed α-synuclein fibril-seeded models attenuate α-synuclein aggregation in neurons (*22*). Conversely, in vitro studies show that nanospheres composed of polyethylenimine-coated carboxyl-modified polystyrene nanoparticles can increase α-synuclein nucleation (*21*). Other types of particle surfaces, such as silica or zirconium, have primary effects on α-synuclein fibril elongation and are essential components of diagnostic α-synuclein seeding assays used to detect aggregated α-synuclein seeds in patient biofluids and tissues (*23*, *24*).

With the rise of nanoparticles in the environment, studies in model systems related to α-synuclein pathobiology may provide insights into what types of nanoparticles to further explore as potential toxins in PD. Among different types of plastic pollutants, the increase in polystyrene nanoplastics in the environment from single-use plastics, recent detection of polystyrene contaminants in blood, and past reports of anionic polystyrene plastics disrupting and crossing the blood-brain barrier suggest polystyrene nanoplastics as a relevant type of particle to explore for interaction with α-synuclein. Here, we find that anionic polystyrene nanoplastics bind to α-synuclein with low to subnanomolar affinity, which rapidly promotes α-synuclein fibril formation and fibril-seeded pathology in cultured neurons, as well as in dopaminergic neurons in the intact wild-type mouse brain. Both α-synuclein fibrils and nanoplastic particles internalize in neurons in a clathrin-dependent manner and converge at the lysosome where new α-synuclein inclusions form. Although a rare occurrence in mice, direct nanoplastic exposure, by itself, can result in the de novo formation of mature phosphorylated α-synuclein inclusions in dopaminergic neurons in the substantia nigra. These results highlight an emergent potential toxin in anionic nanoplastics that might be explored further to better understand a possible role in PD risk and progression.

## RESULTS

### Anionic nanoplastic contaminants catalyze α-synuclein aggregation

**Fig. 1.**
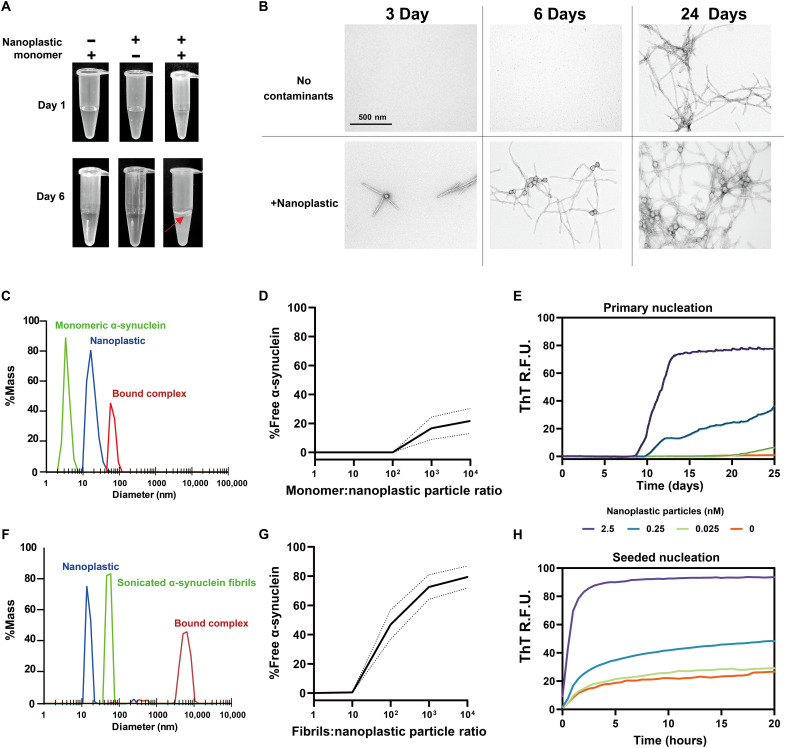
Nanoplastic contaminants complex with monomeric α-synuclein at low stoichiometry to accelerate both spontaneous and seeded fibrillation in vitro. (**A**) Representative appearance of incubation products in centrifuge tubes with 70 μM α-synuclein monomer, with or without 1 nM nanoplastic particles mixed for 1 or 6 days with shaking at 37°C. Red arrow highlights turbidity and a white precipitating layer found with α-synuclein mixed with nanoplastics after 6 days. (**B**) TEM of the products of α-synuclein incubations with or without nanoplastic contaminants incubated for 3, 6, and 24 days. Scale bar, 500 nm. (**C**) DLS profiles plotted to mass distributions for monomeric α-synuclein (green curve) and nanoplastic contaminants (blue curve). Products are from incubations [30 min, room temperature (RT)] at a molar ratio of 10:1 (protein to plastic, red curve). (**D**) Calculated free monomeric α-synuclein at different stoichiometries with nanoplastic after incubation (30 min, RT). Curve is mean with 95% confidence interval (CI) as dashed lines. (**E**) Spontaneous aggregation of α-synuclein (70 μM monomer) at 37°C with shaking in PBS assessed through ThT fluorescence over time with increasing concentrations of nanoplastic contaminants. Each curve is the mean of relative fluorescence units (R.F.U.) from six technical replicates repeated in three independent reactions. (**F**) Representative DLS profiles that include small α-synuclein fibrils (green curve; see fig. S1 for characterization of fibrils). Incubations (30 min, RT) of α-synuclein fibrils with nanoplastic are at a molar ratio of 10:1 (protein to plastic, red curve). (**G**) Calculated free α-synuclein fibrils at different stoichiometries with nanoplastics after incubation (30 min, RT). Curve is mean with 95% CI as dashed lines. (**H**) ThT fluorescence over time, starting with 64 pM α-synuclein fibril particles and 70 μM monomer supplemented with the indicated concentration of nanoplastic contaminants. Each curve represents a mean of six replicates from three independent reactions.

Sources and types of nanoplastics used in this study, along with empirically derived measurements of particle diameters, are indicated in table S1. To determine the effect of polystyrene nanoparticles on the aggregation of wild-type human α-synuclein, we mixed a high concentration of α-synuclein monomeric protein (i.e., ~1 mg/ml), free of high-molecular-weight aggregates (fig. S1, A and B), with 1 nM of ~39.5 ± 0.7 nm average diameter nanoplastics. This concentration of monomeric α-synuclein (~1 mg/ml, endotoxin-free) did not spontaneously aggregate on its own before ~10 days of shaking at 37°C in phosphate-buffered saline (PBS) at pH 7.4, consistent with past reports (*24*, *25*). Cellular concentrations of α-synuclein protein in neurons are reported to be near 50 μM (*26*). Continuous shaking of α-synuclein with the nanoplastic contaminants produces hazy white foamy interfaces within a few days and an overall turbid appearance by 6 days (Fig. 1A). Inspections of products in the solutions via negative-stain transmission electron microscopy (TEM) reveal the presence of multiple α-synuclein fibrils emanating from single plastic particles in as early as 3 days (Fig. 1B). After 24 days of shaking, the overall composition of α-synuclein fibrils under TEM is similar between nanoplastic-contaminated and nanoplastic-free reactions. To confirm that environmentally derived polystyrene nanoplastic, which differs in surface coarseness from smoother engineered polystyrene nanoplastics (*7*), has a similar effect, we produced nanoplastics from single-use red Solo-brand polystyrene plastic cups following National Institute of Standards and Technology (NIST) guidelines (*27*, *28*). These particles, with a size distribution of 115.6 ± 34.3 nm diameter, have a similar nucleating effect on α-synuclein fibrils (fig. S2). In contrast to the effects of anionic nanoplastics, charged graphene nanoparticles used in similar assays have the opposite effect in inhibiting aggregation (*29*), suggesting some level of specificity in how nanoparticles of different compositions interact with α-synuclein.

To further explore the interaction between α-synuclein and nanoplastics, we created binding assays using dynamic light scattering (DLS) to measure interactions between particles. The titration of α-synuclein protein with a set amount of nanoplastic shows that one nanoplastic particle can form a stable complex with more than 100 α-synuclein monomers within minutes at room temperature (Fig. 1, C and D). Evaluation of different concentrations of nanoplastics with respect to a fixed concentration of α-synuclein in real-time quaking induced conversion (RT-QuIC) assays shows that low to subnanomolar concentrations of nanoplastic can nucleate the formation of α-synuclein fibrils (Fig. 1E).

Previous studies using polyethylenimine-coated carboxyl-modified polystyrene nanoparticles showed a primary effect on the nucleation of α-synuclein fibrils, with less of an effect on the seeded growth of fibrils (*21*). Binding assays show that nanoplastics also interact with preformed short α-synuclein fibril seeds (fig. S1, A and B) within minutes of incubation at room temperature, at least ~10 fibril particles per nanoplastic particle (Fig. 1, F and G). In fibril-seeded nucleation reactions in aggregation assays, the anionic nanoplastic particles accelerate new fibril growth at low to subnanomolar concentrations (Fig. 1H). The interaction is charge dependent, as the addition of 400 nM sodium chloride to the solution before incubation of nanoplastic with α-synuclein blocks interactions (fig. S1C). These findings suggest that anionic nanoplastic particles interact with both monomeric and fibrilized forms of α-synuclein in a potentially electrostatic-dependent manner.

### Anionic nanoplastic bind the α-synuclein amphipathic and NAC domains to accelerate α-synuclein aggregation

**Fig. 2.**
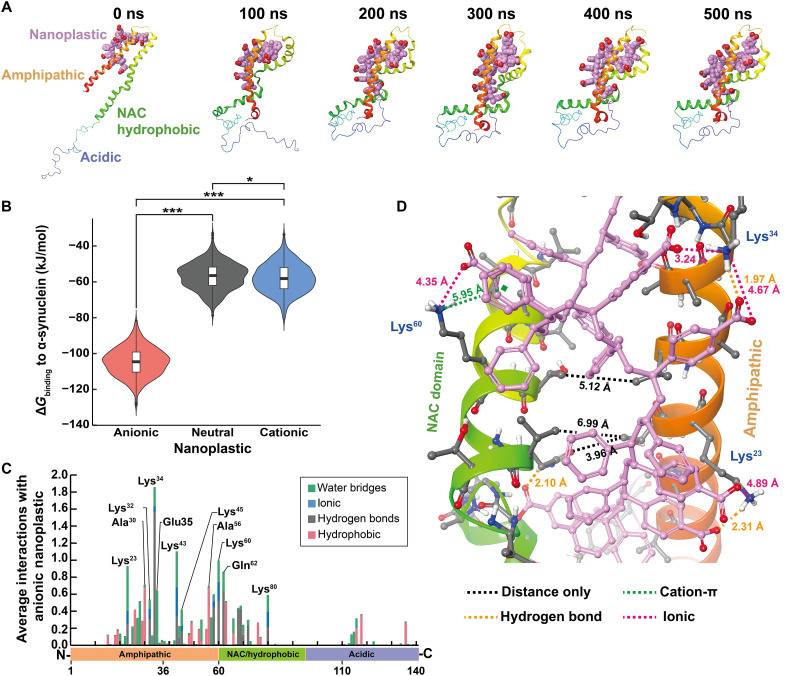
In silico modeling identifies a strong interaction between the amphipathic and NAC domains of α-synuclein and anionic nanoplastic. (**A**) Snapshots at 100-ns intervals of anionic nanoplastic with monomeric α-synuclein across MD simulations. Key domains of α-synuclein are indicated at 0 ns. Anionic nanoplastics are space-filled showing polar hydrogens only; pink = carbon, red = oxygen. α-Synuclein monomers are depicted as ribbons colored by residue index. (**B**) Violin plots overlaid with box-and-whisker plots representing the distribution of Δ*G*_binding_ for each nanoplastic:α-synuclein complex from every 10th frame starting from 200 ns (*N* = 601 each group), time at which each complex converged. See fig. S3 for more MD time frames. *P < 0.05 and ***P < 0.001, one-way ANOVA with Tukey’s post hoc test. (**C**) Histogram demonstrating the average number of interactions between α-synuclein and anionic nanoplastics across converged MD simulation (200 to 500 ns), categorized by four intermolecular interaction types. Stacked bars indicate the sum of all interactions normalized to the length of the trajectory. (**D**) Representative image of anionic nanoplastic bridging the amphipathic and NAC domains of α-synuclein. Distances between the amphipathic and NAC domains are depicted with black dashed lines. Intermolecular forces and their distances between anionic nanoplastic and α-synuclein are indicated. Anionic nanoplastic, depicted as ball and stick, shows polar hydrogens only; pink = carbon, red = oxygen. α-Synuclein (ribbons) are colored by residue index; white = hydrogen, gray = carbon, blue = nitrogen, red = oxygen.

Nanoplastic particles are well known to passively and nonspecifically (weakly) adsorb proteins (*30*, *31*). In contrast to a nonspecific interaction, molecular dynamic (MD) simulations suggest a stable α-synuclein and anionic nanoplastic complex characterized by strong electrostatic attraction and compaction of the amphipathic domain and the adjoining non-amyloid component (NAC) domain (Fig. 2A). A similar complex did not form when the anionic nanoplastic was substituted for neutral and cationic nanoplastics (fig. S3, A to E). Likewise, calculated Δ*G*_binding_ distributions indicate stronger associations with anionic than with neutral or cationic nanoplastics (Fig. 2B and fig. S3, F and G). Being the amphipathic and NAC hydrophobic domains of α-synuclein are rich in positively charged lysine residues, we investigated further whether α-synuclein aggregation results from electrostatic complementarity. MD simulations reveal that key hydrogen bonds and ionic intermolecular interactions drive anionic nanoplastics to specifically bind to residues in the amphipathic and NAC domains (Fig. 2C). The stacked bars in Fig. 2C indicate the sum of all interactions normalized to the length of the trajectory. For example, Lys^80^ has an average of 0.58 nanoplastic interactions, indicating that it interacts with the nanoplastic 58% of the time. Thus, both the amphipathic and NAC hydrophobic domains of α-synuclein form strong interactions with anionic nanoplastic in the model.

Upon closer examination of the predicted complex, the anionic nanoplastic displaces water and intercalates between the amphipathic and NAC domains of α-synuclein, potentially a key step in the eventual formation of β sheets found in fibrils. Not only are the carboxylate groups able to form favorable polar and ionic interactions but also the backbone of the polystyrene nanoplastic allows for good hydrophobic contact across the protein’s surface between the carboxylate and ammonium pairs. Highlighted protein-ligand interactions (Fig. 2D and fig. S4, A and B) are predicted to be essential for the anionic nanoplastic binding to α-synuclein, providing a potential nucleation site for α-synuclein β-sheet stacking. Binding is reminiscent of some models for the α-synuclein amphipathic domain interacting with different phospholipid compositions that also facilitate the eventual formation of β sheets between NAC domains (*32*, *33*), depending in part on electrostatic interactions. These results are indicative of specific binding of anionic nanoplastic with α-synuclein.

Interactions between nanoplastic and α-synuclein protein fibrils were not evaluated in the same model because the structure of the amphipathic domain in α-synuclein fibrils has not been defined according to the latest atomic models, although a similar interaction between α-synuclein and the accessible (e.g., flexible) amphipathic domain in α-synuclein fibrils are expected.

**Fig. 3.**
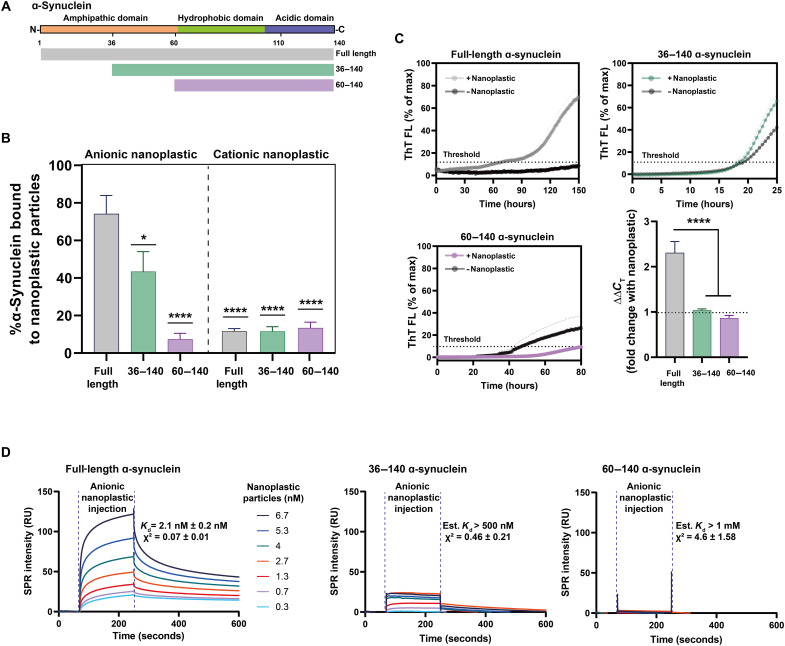
Anionic nanoplastic tightly binds to the α-synuclein amphipathic domain to initiate fibrillation in vitro. (**A**) Domain structure of α-synuclein. Aligned truncated variants in green (36 to 140) and purple (60 to 140) are aligned to full-length protein in gray (1 to 140). (**B**) Column graphs showing the percentage of full-length or truncated monomeric α-synuclein binding to nanoplastic particles (anionic or cationic). DLS resolves complexes at a stoichiometry of 10:1 protein to nanoplastics. Bars represent mean values of 20 independent acquisitions (SEM shown). *P < 0.01 and ****P < 0.0001, one-way ANOVA with Dunnett’s post hoc test. (**C**) Spontaneous full-length or truncated α-synuclein aggregation assessed through ThT fluorescence over time, with or without 2.5 nM anionic nanoplastic particles as indicated. Curves represent mean values from quintuplicate independent reactions (SEM shown). Black dashed horizontal line indicates 10% ThT maximum fluorescence used to calculate threshold (*C*_T_). Bar graph shows mean fold change from control with nanoplastic addition (ΔΔ*C*_T_). Means are from quintuplicate independent reactions (SEM shown). ****P < 0.0001 from one-way ANOVA with Tukey’s post hoc test. (**D**) Representative SPR sensorgrams with full-length α-synuclein (1 to 140, left), truncated 36 to 140 (middle), or 60 to 140 (right), with a range of nanoplastic contaminants in solution. Mean resonance units (RU) in sensorgrams are globally fit to heterogeneous ligand models repeated in triplicate for each protein variant. Average *K*_d_ and χ^2^ values (1 to 140, full-length protein) or estimated values (low-confidence χ^2^ for the truncated proteins) are shown from three independent array runs (SEM indicated).

To investigate further whether the amphipathic domain of α-synuclein is responsible for the interaction with anionic nanoplastic and the promotion of α-synuclein aggregation, we created two truncated α-synuclein proteins, one with half of the amphipathic domain removed and the other with the entire domain deleted (Fig. 3A). These proteins were purified to a high extent and confirmed to be monomeric (fig. S5, A to C). Our results show that without the amphipathic domain, the truncated proteins fail to interact with the anionic nanoplastic (Fig. 3B). These results support the molecular model, indicating that anionic polystyrene’s electrostatic complementarity paired with good hydrophobic contact is driving specificity for α-synuclein and, therefore, nucleation. In addition, cationic polystyrene nanoplastic did not bind to the full-length α-synuclein or the truncated proteins lacking the amphipathic domain. To investigate whether nanoplastic could promote α-synuclein nucleation in the absence of the amphipathic domain, we conducted more aggregation assays. Although the truncated variants aggregate faster than the full-length α-synuclein protein, the addition of anionic nanoplastic particles did not stimulate their aggregation (Fig. 3C), indicating the necessity of the amphipathic domain both for binding and for interaction in aggregation.

These results suggest a possible strong interaction between anionic nanoplastic and the amphipathic domain of α-synuclein. To test this prediction, we used surface plasmon resonance (SPR; fig. S6, A to C) to measure interaction strength between the nanoplastics and α-synuclein. Full-length human α-synuclein monomer has an exceptionally high affinity of ~2 nM dissociation constant (*K*_d_) with anionic nanoplastic at physiological buffer and pH conditions (Fig. 3D). However, binding estimates could not be obtained accurately for α-synuclein affinity to nanoplastics when the protein lacks part or all of the amphipathic domain, or for full-length human α-synuclein with cationic nanoplastic, ostensibly due to relatively weak interactions (fig. S6). Affinity between nanoplastics and full-length α-synuclein fibrils further increases to ~0.03 nM *K*_d_, characteristic of an exceptionally strong interaction with aggregated α-synuclein (fig. S6F). Overall, these findings argue against a passive and nonspecific adsorbance interaction between α-synuclein and nanoplastics. Tight binding of α-synuclein to nanoplastics might drive domain rearrangements that favor the formation of protein fibrils that also have high affinity for nanoplastics.

### Anionic nanoplastics contaminants accelerate fibril-seeded α-synuclein pathology in neurons

**Fig. 4.**
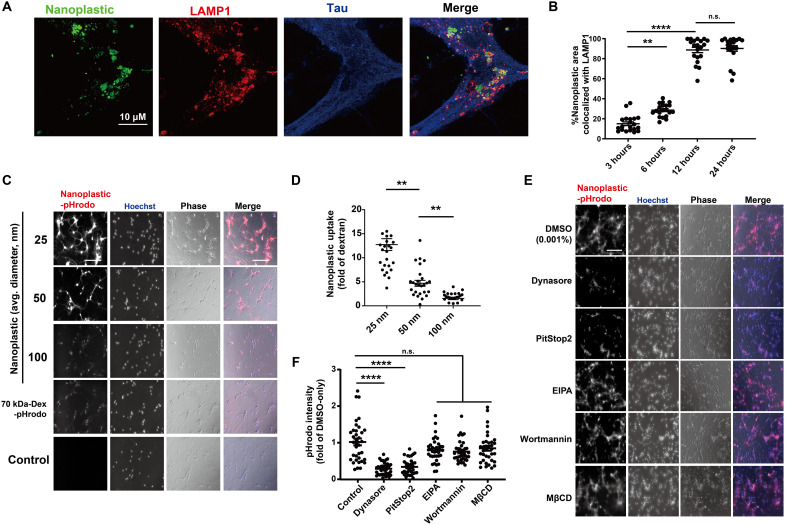
Primary mouse hippocampal neurons endocytose nanoplastics through a dynamin-dependent process. (**A**) Representative Airyscan images from mouse primary hippocampal neurons (DIV 7) treated with FITC-labeled (green) nanoplastics (1 nM) for 12 hours. Neurons were coimmunostained for tau (blue) and the lysosome marker LAMP1 (red). Scale bar, 10 μm. (**B**) Quantification of the percentage of FITC-positive areas (i.e., nanoplastic) in the Airyscan images colocalized with LAMP1. Each dot in the graph represents the mean value of colocalization found within one isolated cell, with at least 20 cells analyzed per group from three independent experiments. Representative images from this analysis are in fig. S8.(**C**) Nanoplastic particles of different diameters (pHrodo-Red, table S1) or 70-kDa dextran (pHrodo-Red labeled) were added (10 μg/ml) to mouse hippocampal neurons (DIV 7) for 2 hours. Representative wide-field fluorescence images are shown with phase-contrast overlays (blue and gray in merges, respectively). (**D**) Quantification of plastic particle uptake, normalized as a fold of pHrodo-Red fluorescent intensity from control pHrodo–70 kDa–dextran. Each dot represents the mean value of one image analyzed from three independent neuronal cultures. (**E**) Primary neurons treated with vehicle (0.001% DMSO) or the indicated endocytosis inhibitor for 20 min before the addition of 1 nM pHrodo-Red nanoplastic particles for 2 hours before imaging. Inhibitors did not affect lysosomal acidification (fig. S9). Hoechst and phase-contrast overlays (blue and gray in merge, respectively) are shown. (**F**) Uptake of nanoplastic particles calculated as a fold of pHrodo-Red fluorescent intensity from DMSO-only treatment. Each dot represents the mean value of one image analyzed from three independent neuronal cultures. All scale bars, 200 μm. In (B), (D), and (F), black bars show group means (SEM indicated) with significance assessed by one-way ANOVA and Tukey’s post hoc test. **P < 0.01 and ****P < 0.001; n.s., not significant.

The mechanisms of neuronal uptake of anionic nanoplastic contaminants has not been extensively studied in the past, but the uptake of small exogenous α-synuclein fibrils into neurons has been reported as dynamin-1 dependent (*34*). Latex-type microspheres (e.g., retrobeads) are often used as retrograde tracers in the brain and other organs. Nanoplastics internalize into NeuN-positive mature neurons [day in vitro (DIV) 7 mouse primary hippocampal neurons] within a few hours (Fig. 4A). Neurons composed more than 70% of the cells in the cultures (fig. S7, A and B). Within 12 hours after uptake, most of the particles in tau-positive cells (e.g., neurons) localize to LAMP1-positive vesicles (e.g., lysosomes; Fig. 4B and fig. S8, A to C). To understand the endocytotic uptake mechanism related to anionic nanoplastic internalization in these neurons and the potential intersection with α-synuclein in the endolysosomal compartment, we incubated neurons with nanoplastics of different sizes (Fig. 4, C and D and table S1) and with inhibitors of different types of endocytosis (Fig. 4, E and F, and fig. S8, D and E). The clathrin-dependent endocytosis blockers Dynasore and PitStop2 (*35*, *36*) effectively impair the internalization of nanoplastics into neurons (Fig. 4, E and F). In contrast, control dextran particle internalization is unaffected by these drugs, because dextran internalization occurs in neurons primarily via fluid-phase endocytosis (*37*). The pH-sensitive dye labeling (i.e., pHrodo-Red) of the nanoplastics helps exclude the possibility of recording colocalization signals from noninternalized particles in the confocal sections, and neither the endocytosis inhibitors nor the nanoplastics had effects on endolysosomal acidity that might otherwise obscure fluorescence intensities (fig. S9, A and B). These results suggest that neurons (at least mature hippocampal neurons in culture) can rapidly internalize charged nanoplastics of different sizes largely through a clathrin-mediated mechanism.

**Fig. 5.**
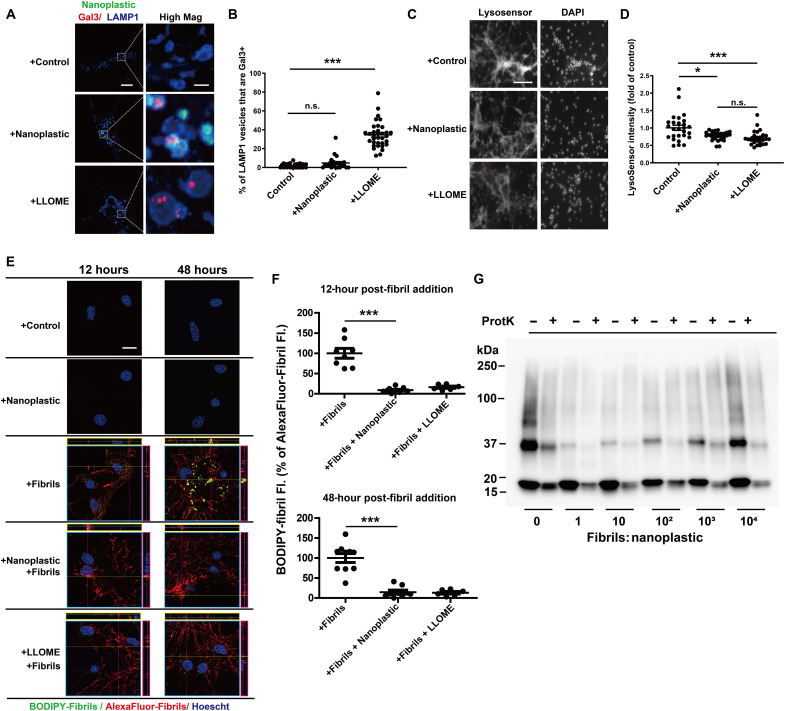
Anionic nanoplastics cause mild lysosomal impairment in neurons and attenuate the degradation of exogenous α-synuclein fibrils. (**A**) Representative Airyscan images of neurons (DIV 7) treated with FITC-labeled nanoplastic (1 nM) or 1 mM LLOME for 24 hours before immunostaining for Gal3 (red) and LAMP1 (blue). Scale bars, 10 and 1 μm (for “High Mag”). (**B**) Colocalization between Gal3 and LAMP1 in vesicles. Each dot represents the mean value of one cell analyzed from at least 20 cells from three independent experiments. (**C**) Representative fluorescence images of live (DIV 7) neurons, first treated with nanoplastic (1 nM) or LLOME (1 mM) for 24 hours, and then stained with 1 μM LysoSensor (green) for 20 min, washed, and stained with Hoechst dye. Scale bar, 200 μm. (**D**) Quantification of relative LysoSensor green signal. Twenty-seven images from three independent experiments were quantified for each condition, with each dot representing results from one image. (**E**) Representative Airyscan images of primary neurons (DIV 7) treated with nanoplastic (1 nM), α-synuclein fibrils (66.7 pM, 1:3 BODIPY-fibrils to Alexa Fluor 647–fibrils), or LLOME (1 mM) for the indicated time (12 or 48 hours). Orthogonal views of sequential z-stacks are shown. Pink box = *y*,*z* plane; yellow = *x*,*z* plane. Scale bar, 10 μm. (**F**) BODIPY-signal (yellow) is calculated as a percentage of total Alexa Fluor 647 fibril signal, representing normalized degraded α-synuclein fibrils. Each dot represents the mean analysis of one image, from three independent experiments, with at least 25 cells analyzed per group. (**G**) Representative immunoblot of denatured total α-synuclein products before and after partial proteinase K digestion, using α-synuclein antibody MJFR1. In (B), (D), and (F), black bars show group means (SEM). **P < 0.01 and ***P < 0.005, from one-way ANOVA with Tukey’s post hoc test.

Some past reports suggest that nanoparticles composed of silica may have adverse effects on lysosome function in neurons and the clearance of amyloid (*38*). As opposed to treatments with the lysosome-damaging agent l-leucyl-l-leucine methyl ester (LLOME), nanoplastic exposure does not cause the accumulation of the lysosomal damage marker galectin3 (Gal3) on LAMP1 vesicles in neurons (Fig. 5, A and B). However, reduced LysoSensor fluorescence observed for both nanoplastic and LLOME treatments might be indicative of a mild lysosomal impairment (e.g., deacidification; Fig. 5, C and D). As the lysosome has been described as the principle degradative organelle in neurons that metabolizes both endogenous low-molecular-weight α-synuclein and α-synuclein aggregates (*39*–*42*), we created a BODIPY-labeled dye-quenched α-synuclein fibril tool [i.e., DQ-α-synuclein] to measure the degradation of α-synuclein at the neuronal lysosome. Fluorescence of the DQ-α-synuclein fibrils only occurs with proteolytic digestion. Consistent with the effects of LLOME exposures, nanoplastics attenuate the degradation of α-synuclein fibrils in neurons both shortly after uptake and localization (at 12 hours) to LAMP1-positive vesicles and at extended time points after internalization (48 hours; Fig. 5, E and F). Despite the effects on lysosome and α-synuclein degradation impairment, there are no reductions observed in the numbers of NeuN-positive cells in culture with nanoplastic treatments (fig. S10A), loss of tau neuronal processes (fig. S10B), or increases in nuclear permeability (i.e., toxicity; fig. S10, C and D). Therefore, nanoplastic exposure at these concentrations appears well tolerated, despite the mild lysosomal impairment. Incubation of α-synuclein fibrils with proteinase K in vitro results in their degradation over time, observed through the loss of higher-molecular-weight proteins resolved by SDS–polyacrylamide gel electrophoresis (SDS-PAGE) and immunoblot (*43*). The addition of different concentrations of nanoplastics to digestion reactions in vitro does not prevent the degradation of α-synuclein fibrils by proteinase K (Fig. 5G), suggesting that nanoplastic binding itself does not provide protection from proteases. These data suggest that nanoplastic impairment of the lysosome may be the principal factor in slowing the degradation of α-synuclein aggregates.

**Fig. 6.**
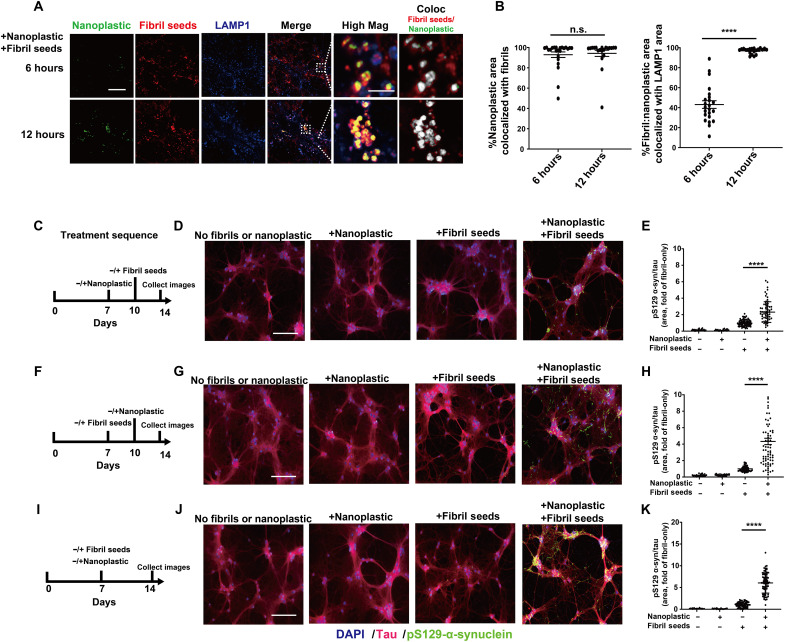
Anionic nanoplastic particles colocalize with α-synuclein fibrils in lysosomes and accelerate pS129–α-synuclein inclusion formation in neurons. (**A**) Representative Airyscan images from mouse primary hippocampal neurons (DIV 7) exposed to both FITC-labeled nanoplastics (1 nM) and Alexa Fluor 647–labeled human α-synuclein fibrils (66.7 pM). Fibrils and nanoplastic particles were not mixed before addition to the cell culture medium. Neurons were immunostained at indicated time points. Colocalized pixels are shown in High Mag in grayscale. Scale bars, 10 and 1 μm (for High Mag). Images from fibrils or nanoplastics at different concentrations individually added are in fig. S10. (**B**) Column graphs showing the quantification of the percentage of nanoplastic area co-positive with fibrils over time (left), and the percentage of nanoplastic-fibril double-positive areas that are colocalized with LAMP1 signal (right). (**C**) Timeline for the sequential addition of unlabeled nanoplastic (1 nM) and then unlabeled fibrils (66.7 pM), and (**D**) representative immunofluorescence for tau and pS129–α-synuclein. (**E**) Quantification pS129–α-synuclein area occupying tau-positive area. (**F**) Reversed addition of particles, first with fibril seeds (DIV 7) and then nanoplastic (DIV14), with (**G**) representative immunofluorescence and (**H**) quantification of pS129–α-synuclein area. (**I**) Simultaneous addition of both fibril seeds and nanoplastic at DIV 7, with (**J**) representative immunofluorescence and (**K**) quantification of pS129–α-synuclein area. All timelines included the independent analysis of no fibrils or nanoplastic added (first column in each graph), as well as the effects of each particle on their own (second and third columns in each graph). Scale bars, 200 μm. Each dot in each column graphs represents the mean value of one cell analyzed from at least 20 cells per condition from three independent experiments. Black bars show group means (SEM), with significance assessed by one-way ANOVA with Tukey’s post hoc test. ****P < 0.0001.

**Fig. 7.**
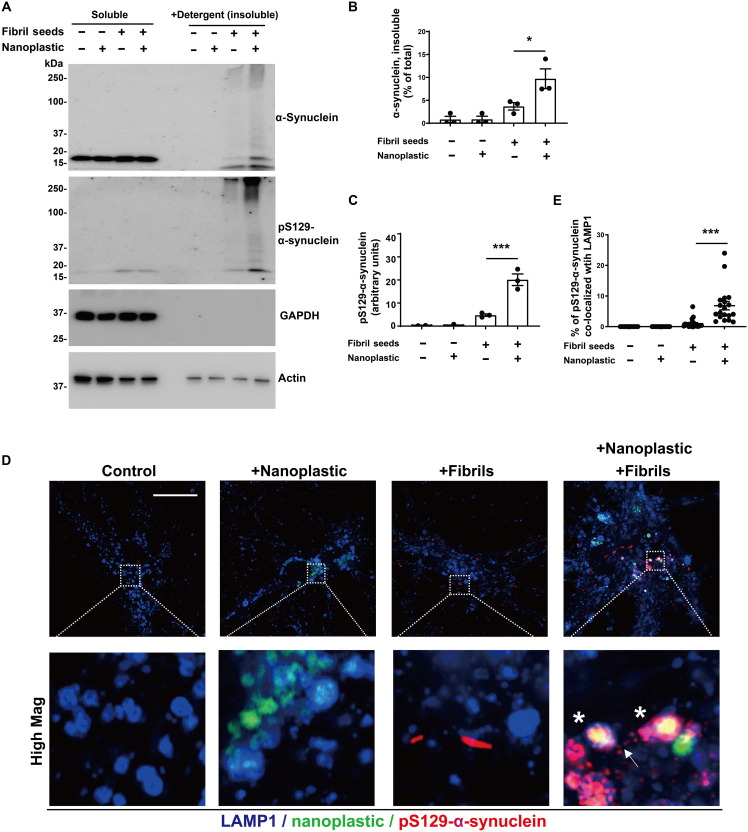
Nanoplastic contaminants enhance the formation of insoluble pS129–α-synuclein at the lysosome in neurons. (**A**) Primary mouse hippocampal neurons (DIV 7) from nontransgenic mice were treated with 1 nM unlabeled nanoplastics together with α-synuclein fibril seeds as indicated. Cells were lysed (DIV 14), and proteins were separated into soluble fractions and SDS-solubilized (i.e., insoluble) fractions, followed by SDS-PAGE and immunoblotting. (**B**) Quantification of the percentage of α-synuclein measured in the insoluble fraction or (**C**) the relative amount of pS129–α-synuclein in the insoluble fraction. Each dot in the columns represents the analysis of an independent lysate from an independent experiment. (**D**) Representative Airyscan images of neurons stained for LAMP1 (blue), nanoplastic (green), and pS129–α-synuclein (red). Asterisks highlight vesicles positive for these markers. Arrow shows pS129–α-synuclein outside of LAMP1 vesicles. Scale bar, 10 μm. (**E**) Quantification of the percentage of pS129–α-synuclein signal that overlaps with LAMP1 in the indicated conditions from (D). Each dot in the column graphs represents the mean value of one cell analyzed from at least 20 cells from three independent experiments. Bars and lines in (B), (C), and (E) show group means (SEM), with significance assessed by one-way ANOVA and Tukey’s post hoc test. *P < 0.05 and ***P < 0.001.

Because both α-synuclein fibrils and anionic nanoplastic uptake depend on dynamin-dependent endocytosis, we investigated whether these particles converge at the endolysosome to increase α-synuclein aggregation. We have previously titrated different concentrations of human α-synuclein fibril particles on primary neurons derived from nontransgenic mice, concluding that the addition of fibrils in the range of 10 to 100 pM induces robust pS129–α-synuclein pathology in as little as 1 week (*44*). Combining labeled (Alexa Fluor 647) α-synuclein fibrils (66.7 pM fibril particles or ~1 μg/ml of α-synuclein) with labeled [fluorescein isothiocyanate (FITC)] anionic nanoplastic results in the colocalization of nanoplastic and fibrils to LAMP1-positive vesicles in neurons within 12 hours (Fig. 6, A and B). In testing different concentrations of nanoplastics on neurons, a modest concentration (1 nM nanoplastic particles or ~10 μg/ml of nanoplastic) increases pS129–α-synuclein pathology compared to neurons treated with only α-synuclein fibrils, whereas lower nanoplastic concentrations (e.g., 10 pM) do not affect fibril-seeded inclusions within this time frame (fig. S10, E to G). The introduction of 1 nM unlabeled nanoplastic particles before the introduction of α-synuclein fibrils, at the same time as fibril addition, or 3 days after fibril addition, increases the amount of pS129–α-synuclein pathology at DIV 14 (Fig. 6, C to K). Potentially consistent with these observations, nanoplastic addition does not noticeably change the uptake of pHrodo-labeled α-synuclein fibrils (fig. S11, A and B). No immunohistochemical signals for pS129–α-synuclein could be resolved with nanoplastic-only conditions or vehicle controls. Biochemical evaluation of lysates from these treatments demonstrates that nanoplastics increase the deposition of α-synuclein into insoluble protein fractions and the proportion of pS129–α-synuclein to total α-synuclein protein (Fig. 7, A to C). High-resolution imaging shows that nanoplastic-induced pS129–α-synuclein pathology significantly increases the proportion of pathology that localizes to lysosomes (Fig. 7, D and E). However, the amount of pS129–α-synuclein pathology now at the lysosome with nanoplastic still represents the minority of overall recorded aggregates, suggesting typical intracellular spread of fibrils out of the endolysosomal compartment as expected in this model. Overall, these results indicate that anionic nanoplastic contaminants have potent (i.e., nanomolar) capacity to promote pS129–α-synuclein pathology in cultured neurons.

### Anionic nanoplastics accelerate seeded pathological α-synuclein spread through the mouse brain

**Fig. 8.**
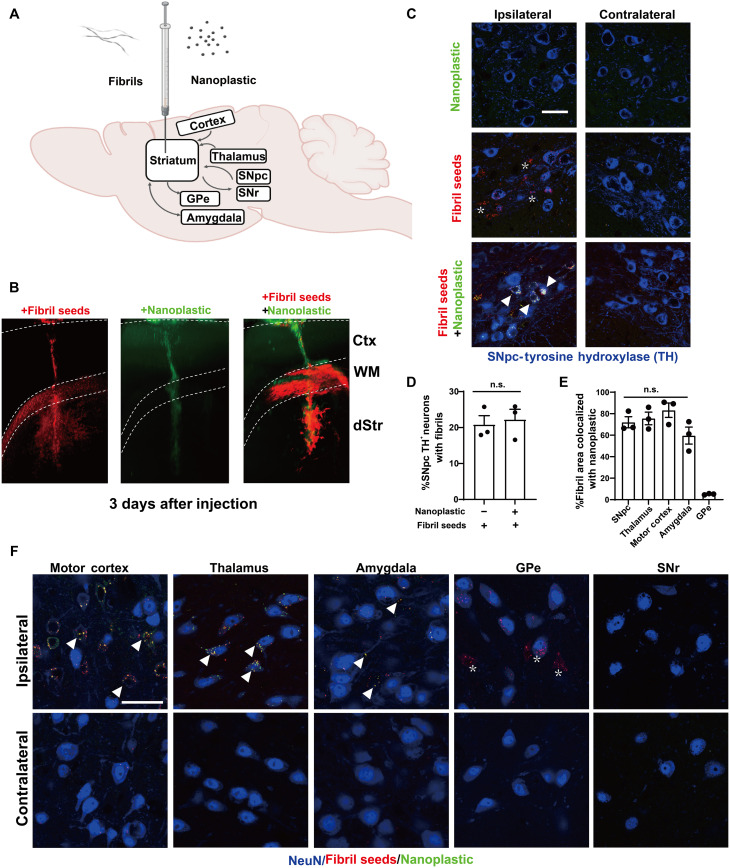
Anionic nanoplastics contaminants traffic with α-synuclein fibrils along retrograde projections and accumulate in neuronal bodies. (**A**) Depiction of ~P90 male (nontransgenic) mice injected unilaterally in the dorsal striatum with 15 μg of FITC-conjugated nanoplastic, or 4.5 μg of Alexa Fluor 647–labeled human α-synuclein fibrils, or the injection of these particles together. Anterograde projections from the injection site include the globus pallidus (GPe) and substantia nigra pars reticulata (SNr), whereas retrograde projection targets include the amygdala, motor cortex, thalamus, and SNpc. (**B**) Representative light sheet microscopy of mouse brain, analyzed 3 days after injections, with α-synuclein fibril-only (red), nanoplastic-only (green), or co-particle injections. The cortex (Ctx), corpus callosum (WM), and dorsal striatum (dStr) layers are indicated. (**C**) Representative Airyscan images of dopaminergic cells in the SNpc, with arrowheads indicating cells with vesicles filled with both fibrils and nanoplastics (white signal). Asterisks show neurons from fibril-only injections. Results are representative of three mice injected per group. Scale bar, 50 μm. (**D**) Column graph showing the mean proportion of dopaminergic neurons positive for fibrils with or without nanoplastic coinjection. (**E**) Column graph showing that the proportion of fibril-nanoplastic co-positivity is similar across retrograde-targeted brain regions, with the exception of the GPe (anterograde from injection site). Each dot represents the mean of sections analyzed through one separate animal; one-way ANOVA with Tukey’s post hoc test was used. (**F**) Representative Airyscan images of neurons analyzed in (E), with arrowheads highlighting neurons filled with vesicles co-labeled (yellow) with nanoplastic and fibrils, whereas asterisks highlight neurons filled with only fibrils (GPe). No fibrils or nanoplastics were detected in the SNr. Scale bar, 50 μm.

Small α-synuclein fibrils travel in both anterograde and retrograde directions away from injection sites in the mouse brain (*45*). Latex microbeads (e.g., acrylic latex) are effective retrograde tracers in neurons and other types of cells (*46*). However, the distribution and trafficking patterns for anionic polystyrene nanoplastic contaminants, once inside the brain, have not been well studied. To explore the distribution of these nanoplastics in the brain after a single intracranial injection into the dorsal striatum, light sheet microscopy and confocal analysis show that labeled (FITC) nanoplastic particles on their own do not spread well from the injection site (Fig. 8, A to C, and fig. S12, A to C). In contrast, α-synuclein fibrils distribute readily, accumulating in neurons in the cortex, thalamus, and amygdala and in dopaminergic neurons in the substantia nigra pars compacta (SNpc). The concentration of fibrils injected (4.5 μg, single site) is an amount known to induce the production of hundreds of pS129–α-synuclein inclusions in these brain regions several months after the injection (*44*, *47*). The co-injection of nanoplastics (15 μg) with α-synuclein fibrils results in a broadening of nanoplastic distribution similar to α-synuclein fibril particles on their own (Fig. 8, D to F), with the noticeable exception of anterograde targets from the injection site [e.g., globus pallidus externa (GPe)]. A lower concentration of 1.5 μg of nanoplastic injected with the fibrils did not yield detectable nanoplastic particles in neurons (fig. S12A), so we prioritized the higher 15-μg nanoplastic injection to test for effects on accumulations in neurons and α-synuclein aggregation.

**Fig. 9.**
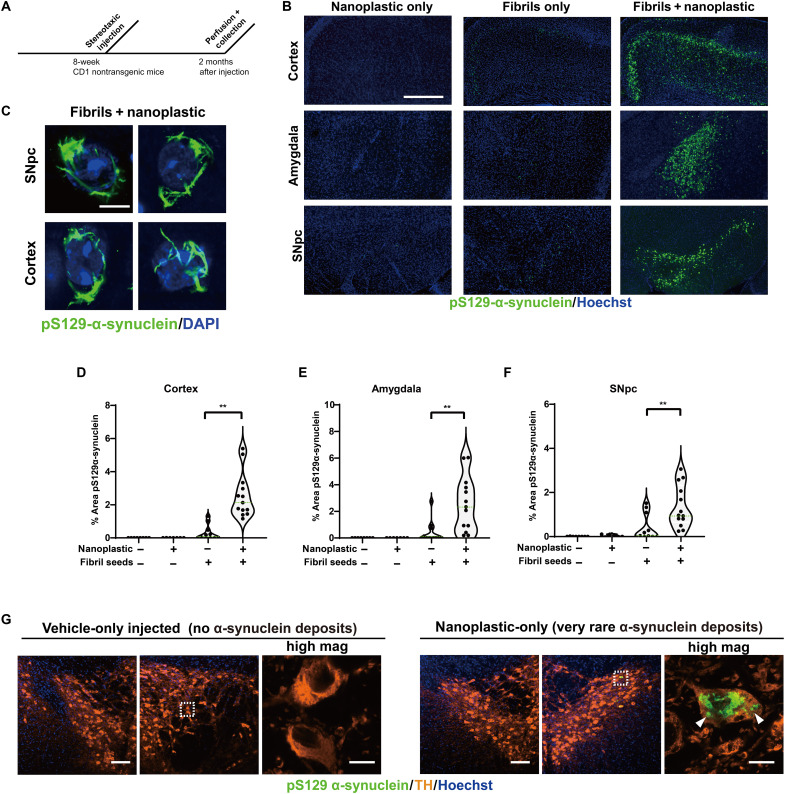
Anionic nanoplastic contaminants in the brain synergize with α-synuclein fibrils to increase the deposition of pathology in the cortex, amygdala, and substantia nigra in nontransgenic mice. (**A**) Timeline for the injection of adult (nontransgenic) male mice with control saline (*N* = 8 mice), 4.5 μg of unlabeled α-synuclein fibrils (*N* = 12 mice), or 4.5 μg of unlabeled α-synuclein fibrils combined with 15 μg of unlabeled nanoplastic (*N* = 12 mice). (**B**) Representative immunofluorescence staining of α-synuclein pathology (pS129–α-synuclein, green) in the indicated brain region 2 months after injections. Scale bar, 0.5 mm. (**C**) Representative high-magnification Airyscan images of pS129–α-synuclein staining inside of dopaminergic neurons (SNpc) or pyramidal neurons in the motor cortex (cortex). Scale bar, 10 μm. (**D**) Violin plots showing the distributed α-synuclein inclusion burden (pS129–α-synuclein area in each image) in ipsilateral cortex, (**E**) amygdala, and (**F**) SNpc. Mean percent areas of the indicated brain nuclei occupied by pS129–α-synuclein staining are plotted, where each dot represents the analysis of all sections through the respective brain region of one animal. **P < 0.01, Mann-Whitney *U* test. (**G**) Two months after nanoplastic-only injections, 3 of 10 mice demonstrated robust but isolated α-synuclein inclusions (green) in dopaminergic neurons in the SNpc, suggesting that nanoplastic on its own can induce de novo mature inclusions. Bounding boxes are magnified to highlight these inclusions (arrowheads). Scale bars, 100 and 10 μm (for “high mag”).

With a co-injection of nanoplastics with α-synuclein fibrils, ~20% of dopaminergic neurons in the SNpc 3 days after injection are positive for both α-synuclein fibrils and nanoplastics in their cell soma, with about 75% of α-synuclein fibril signal colocalizing with nanoplastic (Fig. 8, D and E). On the basis of the robust observed colocalization between nanoplastic particles and α-synuclein fibrils in neurons in the model, and the propensity of nanoplastics to stimulate α-synuclein aggregation, we investigated whether the neurons harboring the nanoplastics might be more susceptible to form more α-synuclein pathology. Similar to a previous report (*45*), human α-synuclein fibrils injected into nontransgenic outbred CD1 mice poorly seeded phosphorylated pS129–α-synuclein–positive inclusions through the cortex, amygdala, and SNpc (Fig. 9, A and B). However, coadministration of nanoplastic together with the fibrils results in the formation of mature cytoplasmic pS129–α-synuclein inclusions in dopaminergic neurons (Fig. 9C) and a large overall increase in pS129–α-synuclein pathology across the cortical mantle, amygdala, and SNpc (Fig. 9, D to F). No pS129–α-synuclein inclusions occurred in vehicle-only injected animals, and no overt neurodegeneration was present that might otherwise confound the analysis of the neuronal pathology (fig. S13). Although a rare occurrence, we noted that 3 of 10 adult male mice injected in the striatum with only unlabeled anionic nanoplastics (no α-synuclein protein) demonstrated several dopaminergic neurons each in the SNpc filled with pS129–α-synuclein pathology (Fig. 9G). These results suggest that anionic nanoplastics might be capable of de novo induction of pS129–α-synuclein inclusions in dopaminergic neurons several months after the plastics are internalized by neurons in the brain.

### Double-shot pyrolysis gas chromatography–mass spectrometry detects styrene ions in human brain tissues

According to Leslie *et al.* (*8*), it is a common occurrence for blood samples to harbor (e.g., >1 μg/ml) polystyrene contamination, and as opposed to other types of plastic pollution, nanoplastic polystyrene particles are known to cross the mammalian blood-brain barrier (*48*). Recently, we reported strong α-synuclein seeding activities observed in α-synuclein aggregation assays in lysates from frozen frontal cortex brain tissues from Lewy body dementia cases (*24*). In adopting the protocol of Leslie *et al.* (*8*) that describes the sensitive detection of styrene fingerprint ions from lyophilized whole-blood samples, frontal cortex brain tissues from Lewy body dementia with strong α-synuclein seeding activity also yield strong styrene ion traces according to a double-shot pyrolysis gas chromatography–mass spectrometry method (fig. S14). The styrene traces are unlikely to be from blood, as larger blood vessels were removed from the cortical brain tissue after lyophilization and before brain tissue pulverization. Furthermore, the brain samples did not contact any polystyrene plasticware during processing (to our knowledge). In all cases, robust styrene ion traces were detected in both control and Lewy body–affected frontal cortex brain tissue, similar to the styrene ion traces previously described from healthy blood samples. However, different from that study, in brain samples, ions from other types of plastic (i.e., polypropylene or polyethylene) were not detected from the brain tissue pyrolysates. It is important to note, as pointed out by others (*8*), that these results are not definitive for the identification of polystyrene nanoplastic, and additional orthogonal methods will need to be developed to confirm the presence and concentrations of potential polystyrene particles in complex tissues like blood and brain. However, these data provide some of the first measurements for these contaminants that might be present as nanoplastics in human brain tissue.

## DISCUSSION

**Fig. 10.**
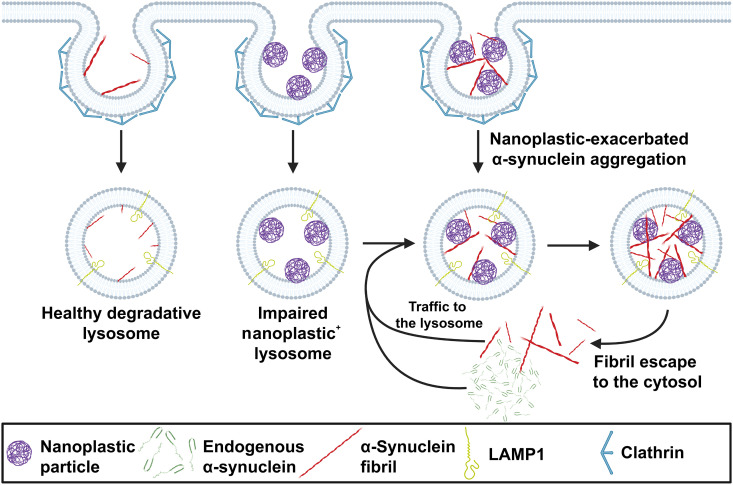
Hypothetical model for the pathological interaction between α-synuclein and nanoplastic contaminants in neurons. Nanoparticles that have the intrinsic capacity to disrupt and cross the blood-brain barrier, such as small charged polystyrene particles, may come into contact with neurons that express high levels of α-synuclein and can harbor aggregates in disease. Peripheral cells susceptible to α-synuclein aggregation and outside of the blood-brain barrier (e.g., vagal neurons) may also come into contact with nanoplastic contaminants. Both α-synuclein fibrils and nanoplastics can enter the endolysosomal compartment in neurons through a clathrin-dependent endocytosis process. A healthy lysosome might fully degrade an α-synuclein aggregate. However, if a lysosome is impaired by nanoplastic particles, α-synuclein aggregates might not degrade and endogenous α-synuclein trafficked to the lysosome may inadvertently spur the formation of new α-synuclein fibrils through the potent pro-aggregation catalytic action of nanoplastics. This feedforward pathway highlights a two-part proposed mechanism whereby nanoplastic contaminants may accelerate the formation of misfolded α-synuclein in the endolysosome compartment and simultaneously impair the degradative action of the organelles (i.e., lysosomes) primarily responsible for their clearance.

Here, our conclusions center on four main observations. First, anionic polystyrene nanoplastics bind the amphipathic domain of human wild-type α-synuclein with very high affinity. Second, α-synuclein binding to nanoplastic particles promotes α-synuclein aggregation. Molecular modeling suggests that this may be due to rearrangement of NAC domain positioning and the displacement of water for negatively charged polymer subunits between the domains. Third, both nanoplastic particles and exogenous α-synuclein fibrils enter neurons in a large part through a clathrin-dependent endocytosis process, whereby both particles converge at the lysosome. Once inside neurons, anionic nanoplastics cause a mild lysosomal impairment that slows the degradation of aggregated α-synuclein and up-regulates the production of fibril-seeded pS129–α-synuclein inclusions. Last, the coadministration of anionic nanoplastic contaminants with exogenous human α-synuclein fibrils markedly up-regulates the spread of pS129–α-synuclein inclusions across interconnected brain regions in nontransgenic mice, including the formation of pathology in dopaminergic neurons in the SNpc. A hypothetical model that graphically summarizes these observations is presented in Fig. 10.

A biological context for these results can be inferred from other recent studies. Microplastic pollution and mammalian exposures to very small plastic particles is likely to increase for the foreseeable future (*49*). This is due to continued environmental degradation of older microplastics, for example, through ultraviolet irradiation that can charge the particles and mechanical shearing that reduces average particle diameters (*50*). Therefore, it is relevant to explore the potential impact these emerging particles might have on disease risk, either risk imposed by particles currently in the environment that are difficult to measure with current technologies or those that likely exist in the foreseeable future. Polystyrene microplastics, derived primarily from pervasive single-use plastics, have been identified in low microgram per milliliter quantities in blood samples from young healthy control subjects and may have the propensity to disrupt and cross the mammalian blood-brain barrier to traverse into deep parenchymal brain tissue (*8*, *11*–*13*, *51*). Among other diseases potentially affected by nanoplastic exposures, PD is considered one of the fastest growing neurological conditions in the world, and emerging toxicants from the environment might affect disease risk in complex ways, for example, in interacting with other genetic susceptibilities (*19*, *52*). The intersection of nanoplastics at the lysosome with α-synuclein in neurons implicates a growing body of evidence that suggests that lysosomal dysfunction may be at the heart of pathogenic mechanisms for PD risk (*53*). While PD has existed long before the rise of nanoplastics in the environment, nanoplastics exposures may nevertheless present a new cumulative risk, depending on lifetime exposure levels, that can be explored in future studies. Here, the robust interaction between α-synuclein and nanoplastics contaminants, coupled with the preliminary detection of polystyrene pollution in the human brain, may provide a rationale for further exploration related to nanoplastic exposures and PD risk.

In vitro, polystyrene contaminants bear a notable capacity at very low concentrations to promote seeding of α-synuclein fibrils from otherwise soluble α-synuclein protein. Interactions were noted with the α-synuclein amphipathic domain that remains exposed in both monomeric protein and highly structured fibrils. While the concentration of native α-synuclein at the synapse in healthy neurons has been estimated, whether or not anionic nanoplastics accumulate in neurons in humans (in health or disease) has not yet been evaluated to our knowledge. A vexing issue in nanoplastic research is that sensitive methods that might characterize the sizes of smaller nanoparticles derived from complex matrices like brain tissues and biofluids do not yet exist, so exposures are difficult to understand. While pyrolysis mass spectrometry methods like those used here for human brain tissue analysis are sensitive to low parts per million, they do not divulge the sizes and exact molecular compositions of the parental polymers. On the basis of what is known in mammalian models, it seems unlikely that larger microplastics would be able to traverse through endothelial barriers and cells composing the blood-brain barrier. However, as α-synuclein seeding activity and pathology have been detected in the periphery including the gastrointestinal system of PD patients, nanoplastic contaminants need not necessarily invade the central brain to potentially corrupt α-synuclein folding to affect disease risk or progression (*29*). Future studies are required to clarify these points.

From several different types of pervasive plastic waste types known, polystyrene nanoplastic was selected here because of the described action at the blood-brain barrier and positive detection in human blood. Other types of pervasive plastic waste such as polyethylenes, polycarbonates, or polypropylene may also interact with α-synuclein (in the brain or periphery) and should be evaluated in future studies. The appearance of sparse but mature phosphorylated α-synuclein inclusions in dopaminergic neurons in the midbrain of a few mice injected with only nanoplastics is a concerning result. According to a prion-like hypothesis for PD, these inclusions might spread to interconnected neurons and propagate through the brain, if given enough time. In humans, the detection of such sparse pathology might be consistent with a pathological diagnosis of incidental Lewy body disease. Some have hypothesized that incidental Lewy body disease is a precursor to PD and other Lewy body diseases (*54*). Further evaluation of plastic exposures with mice bearing additional risk factors for PD that might impair the neuronal lysosome, like advanced age, a *GBAI* mutation, or a *LRRK2* mutation, may be informative.

The combined injection of human α-synuclein fibrils with nanoplastic contaminants highlights a strongly synergistic effect on the formation of inclusions in vulnerable brain regions in the mice. Perhaps, a neuron harboring negatively charged nanoplastic at the acidic lysosome in the cell body would be more likely to develop an α-synuclein inclusion than a healthy lysosome that readily degrades protein aggregates before they can propagate. Together, these initial results in models highlight a potential toxin challenge in charged nanoplastics for further exploration in PD-related mechanisms. Future studies using physiologically relevant nanoplastic exposures, as they continue to be defined in the environment, in combination with long-duration progressive PD-related models, will better inform emergent environmental risks.

## MATERIALS AND METHODS

### Nanoplastics and particle sizing

Table S1 lists the different types of plastic particle catalog numbers used in each experiment in this study. To generate dye-labeled particles, polystyrene nanoplastic particles (at a concentration of 5 mg/ml) carrying amine surface modifications were incubated with pHrodo iFL Red STP ester, amine reactive dye (100 μg/ml) (Thermo Fisher Scientific, catalog no. P36010) in 0.1 M sodium bicarbonate buffer (pH 8.3). Free pHrodo iFL Red STP ester was neutralized using 100 mM tris-HCl at pH 7.4 and removed using Zeba Spin Desalting Columns (Thermo Fisher Scientific, catalog no. 89889). pHrodo-conjugated 10-kDa dextran was from Thermo Fisher Scientific (P10361). To generate nanoplastics with mechanical stress, a custom abrasion machine was used as previously described (*27*). First, a plastic cup with polystyrene indicated as the main component was cut into 2.5-cm^2^ pieces for mechanical abrasion. The sample was attached to the machine sample holder, and a 2-kg weight provided a normal force pressure, while abrasion took place using P80 aluminum-oxide sandpaper (VWR, catalog no. 296102). The sample was pressed against the abradant and rotated using a motor at a constant speed of around 800 to 1000 rpm. The sample was enclosed in a chamber that was under a hosed fume extractor, allowing for abraded microplastics to be collected for further analysis. To further generate nanoplastic particles, 0.5 g of microplastic powder was sonicated using a Fisher Scientific Sonic Dismembrator model 500 and 13-mm diameter probe tip at 30% power, 10-s pulse, 10-s off, for a total of 2 hours. The sonicated microplastic and nanoplastic mixture was passed through a syringe filter with 0.22-μm pore size and concentrated to 200 μl using an Amicon Ultra Centrifugal Filter Unit with 10-kDa molecular weight cutoff membrane (Millipore, catalog no. MPUFC901024).

Particle number and size distribution of nanoplastic contaminants were analyzed using a ZetaView PMX-110 light scattering video microscope (Particle Metrix, Meerbusch, Germany) as previously described (*55*) and a Titan DynaPro (Wyatt Technology) for DLS analysis, at ~25°C. For DLS, the size distribution was analyzed using the Dyna V6.3.4 software package. For all measurements, samples were diluted in PBS (pH 7.4). For ZetaView, tracking measurements were recorded and analyzed for three video cycles at each of 11 positions. Two-second video recordings at a rate of 30 frames per second were taken for each cycle. Video captures were analyzed using ZetaView Software 8.04.02 SP3, with specific parameters set as follows: maximum area: 5000, minimum area: 10, maximum brightness: 25, minimum brightness: 15, tracking radius: 100, minimum trace length: 20, shutter: 100, and sensitivity: 92. The molarities reported for the particles are calculated from the particle number of the different preparations.

### Purification of recombinant human wild-type α-synuclein, truncated variants, and fibrils

Complementary DNA (cDNA) encoding human α-synuclein was synthesized and subcloned into pET21 bacterial expression plasmid. BL21 (DE3-RIL) CodonPlus cells (Agilent, catalog no. 230245-41) were cultured in broth (Thermo Fisher Scientific, catalog no. 10855001) to optical density at 600-nm wavelength of 0.8 at 37°C before supplementation with 0.5 mM isopropyl β-d-1-thiogalactopyranoside to induce α-synuclein expression for an additional 16 hours at 18°C, followed by centrifugation. Cell pellets were lysed in 10 mM tris-HCl (pH 7.6), 0.75 M NaCl, 1 mM EDTA, and 1 mM phenylmethylsulfonyl fluoride (PMSF) and sonicated at 70% power (Fisherbrand, Model 500 Dismembrator) for 1 min. Lysate was incubated in boiling water for 15 min before centrifugation. The supernatant was collected and dialyzed into 10 mM tris-HCl (pH 7.6), 50 mM NaCl, 1 mM EDTA, and 1 mM PMSF before being loaded to a HiPrep Q HP 16/10 Column, 1 × 20 ml (GE Healthcare, catalog no. 29018182) with a low-salt buffer containing 10 mM tris-HCl (pH 7.6), 25 mM NaCl, and eluted with a linear gradient of high-salt buffer [10 mM tris-HCl (pH 7.6) and 1 M NaCl]. Eluted fractions containing single bands for α-synuclein (analyzed through Coomassie staining after SDS-PAGE; fig. S1) were combined, dialyzed with PBS, and concentrated to 15 mg/ml. As endotoxin has a strong effect on α-synuclein fibrillization, endotoxin in the purified recombinant α-synuclein was removed using depletion columns (GenScript, catalog no. L00338). Iterative column passes were performed until the final endotoxin level was <0.1 U/mg of protein, as determined by LAL chromogenic endotoxin quantification (GenScript, catalog no. L00350C).

To generate preformed α-synuclein fibril seeds, 5 mg/ml (~350 μM) of purified human α-synuclein in endotoxin-free PBS buffer was incubated in a tube mixer (Eppendorf, Thermomixer Compact 5350 Mixer) at 37°C with continuous agitation at 800 rpm for the indicated time interval or 5 days. Pelleted and PBS-washed α-synuclein fibrils were sonicated using a cup horn sonicator (Q-Sonica, model Q700) with an active liquid cooling system that maintained the chamber temperature at 10°C. Some experiments required labeled α-synuclein fibrils. To generate Alexa Fluor 647, pHrodo, or BODIPY-FL–conjugated α-synuclein fibrils, α-synuclein fibrils were incubated with 100 μg/ml of Alexa Fluor 647 *N*-hydroxysuccinimide (NHS) ester (Thermo Fisher Scientific, catalog no. A20006), pHrodo iFL Red STP ester (Thermo Fisher Scientific, catalog no. P36010), or BODIPY-FL NHS ester (Thermo Fisher Scientific, catalog no. D2184) in 0.1 M sodium bicarbonate buffer (pH 8.3) for 1 hour at room temperature. Free dyes were neutralized using 100 mM tris-HCl at pH 7.4 and removed through differential centrifugation and extensive washing of the fibril pellets in PBS. Absorbance values corresponding to the free dyes were monitored until values returned to background levels, indicating the complete removal of free dyes. DQ-α-synuclein refers to BODIPY-FL–labeled α-synuclein fibrils that have not been digested by proteases and have little to no associated fluorescence under physiological conditions. Dye-conjugated α-synuclein fibrils were washed at least five times before sonication to generate working fibril seeds.

### α-Synuclein sedimentation and RT-QuIC analysis

Human α-synuclein was mixed with plastic nanoparticles at indicated concentrations in a mixer (Eppendorf, Thermomixer Compact 5350 Mixer) at 37°C with continuous agitation at 800 rpm. Products were separated by sedimentation into heavy insoluble (pellet) and soluble (supernatant) fractions through centrifugation at 150,000*g* (Beckman MAX-XP). Pellets and the supernatants were resuspended into a 2× Laemmli sample buffer (Bio-Rad, catalog no. 1610737EDU) and analyzed by SDS-PAGE followed by Coomassie blue stain. RT-QuIC analysis was performed as previously described (*24*). Human α-synuclein was mixed with plastic as indicated or preformed α-synuclein fibril seeds. Reaction mixtures were supplemented with 10 μM thioflavin-T (ThT; Sigma-Aldrich, catalog no. T3516-25 g) as indicated to detect the β-sheet structure in the forming α-synuclein fibrils, with baseline and background fluorescence subtracted. All reactions were performed in ultralow-binding 384-well plates (Corning, catalog no. 4588) with clear bottoms and sealed with foil (Bio-Rad, catalog no. MSF1001). Fluorescence was monitored on a FLUOstar Omega (BMG) plate reader at 37°C for the indicated time with 60 s of shaking at 700 rpm followed by 60 s of resting. ThT signal was recorded every 30 min at 448 ± 10 nm excitation and 482 ± 10 nm emission. Threshold values (*C*_T_, hours) correspond to the time needed to achieve 10% of the maximum FL intensity (background subtracted).

### In vitro protease digestion assays

Preformed α-synuclein fibril seeds (125 nM) were prepared in the absence or presence of nanoplastic particles at the indicated concentration and incubated at ~25°C for 30 min. Equal amounts of proteinase K (20 nM, Thermo Fisher Scientific, catalog no. 25530015) were added to each reaction that were incubated at 37°C for 10 min. Reactions were quenched through the addition of a 2× Laemmli buffer and boiling at 95°C for 10 min. Reactions were analyzed for remaining α-synuclein reactivity of >17 kDa as resolved by SDS-PAGE. Proteins were transferred to 0.22-μm nitrocellulose membranes, and immunoblotting was performed using anti–α-synuclein antibody (Abcam, catalog no. ab138501).

### SPR analysis

Binding kinetics of the nanoplastic particles were performed using a Biacore T200 SPR biosensor system (Cytiva, catalog no. 28975001). Monomeric full-length α-synuclein and N-truncated variants in solution (35 μM) or sonicated fibrils (64 nM) were conjugated to CM5 sensor chips (Cytiva, catalog no. BR100399) using amine coupling chemistry at a 1:1 ratio of NHS (11.5 mg/ml) and 1-ethyl-3-(3-dimethylaminopropyl)carbodiimide (75 mg/ml). Before immobilization, preconcentration series were conducted with α-synuclein proteins in 10 mM sodium acetate (pH 4.0 to 5.0) to select efficient conjugation conditions for each protein. Activation of the chip was 420 s followed by immobilization steps for 30, 60, and 120 s until an increase in SPR intensity of 200 to 1000 response units was observed. Deactivation of the surface was conducted by adding 1.0 M ethanolamine hydrochloride–NaOH at pH 8.5 at a 12-min contact time with the running buffer “HBS-EP+” (Cytiva, catalog no.BR100669). For kinetic analysis, ligands were injected at a flow rate of 50 μl/min with contact times of 180 s, dissociation times of 600 s, and data collection rates of 10 Hz at ~25°C. Regeneration at the end of the kinetic program was conducted at 50 μl/min for 30 s, with 10 s of stabilization period using 10 mM glycine (pH 2.0) to remove bound particles and normalize the chip for the next cycle. Biacore Evaluation software was applied for kinetics analysis. Sensorgrams were reference-subtracted and analyzed using a heterogeneous ligand binding model to extract kinetic values from triplicate runs.

### Computational approaches

Using Spartan 20 (https://wavefun.com/), an equilibrium geometry calculation was performed on a 20-mer of neutral nanoplastic. The method was as follows: Density-function theory (DFT) = B3LYP 6-311+G**, total charge = 0, unpaired electrons = 0, solvent = conductor-like polarizable continuum model, dielectric = 78.3 (water). The DFT-optimized neutral nanoplastic structure was exported to “SDF” format and transferred to Maestro (Schrödinger Inc.). The System Builder task was launched to generate a solvent boundary for simulation. The method was as follows: solvent model = simple-point charge (SPC), box shape = orthorhombic, box size = buffer, 20 Å × 20 Å × 20 Å, exclude ion and salt placement within 20 Å of ligand, neutralize model by adding Na^+^ or Cl^−^ ions, and add 0.15 M NaCl. The MD task was launched, and the solvated system was simulated for 50 ns using Desmond. The method was as follows: simulation time = 50 ns, recording interval = 50.0 ps, ensemble class = NPT, temperature = 310.15 K, pressure = 1.01325 bar. To produce the anionic nanoplastic, the MD converged/optimized neutral nanoplastic was functionalized at the para-positions of the phenyl rings with carboxylate groups. Adopting a previously reported model (*7*) of anionic nanoplastic, eight carboxylate groups were added across the nanoplastic 20-mer at every second phenyl group functionality. A solvated model of anionic nanoplastic was prepared and simulated using identical parameters for neutral nanoplastic. To produce cationic nanoplastic, the carboxylate groups of anionic nanoplastic were replaced with ammonium groups and also simulated using identical parameters for neutral nanoplastic. Desmond MD jobs were processed on the Duke Compute Cluster.

Human wild-type α-synuclein monomer structure [Protein Data Bank (PDB) 1XQ8] was imported and prepared with the Protein Preparation Workflow task. Preprocessing was performed with the following parameters: fill in missing side chains; assign bond orders, using Combined Chemical Dictionary (CCD) database; replace hydrogens; create zero-order bonds to metals; create disulfide bonds; fill in missing loops using Prime; sample water orientations, use crystal symmetry, minimize hydrogens of altered species, use PROPKA with pH 7.4; restrained minimization was then performed, converging heavy atoms to root mean square deviation of 0.3 Å using OPLS4; waters were removed 3.0 Å beyond het groups. With the protein and nanoplastic ligands prepared, Induced Fit Docking (IFD) was launched to dock the nanoplastics to α-synuclein. The receptor box was centered about residues Met^1^, Val^37^, Lys^45^, and Ala^85^ with a box size of 46 Å (maximum allowed size) to capture the N-terminal region and allow for unbiased docking. Prime refinement was performed to optimize residues within 12.0 Å of the ligand poses. Glide redocking was performed on structures within 30.0 kcal/mol of the best structure but keeping only the top 20 structures overall, using standard precision. Once IFD was completed, solvated models of the nanoplastics with α-synuclein were prepared and simulated using a 10 Å × 10 Å × 10 Å orthorhombic buffer box and a simulation time of 500 ns; all other parameters were identical to those previously described for preparation of the nanoplastics.

For each of the nanoplastic-protein complexes, all frames from the Desmond trajectories were loaded into Visual Molecular Dynamics (VMD) and the final 300 ns (6001 frames) was exported to GROningen format. GROMACS (GROningen Machine for Chemical Simulations) was then used to extract every 10th frame (netting 601 frames) to create a PDB ensemble of each complex (*56*). The resulting complexes were analyzed for Δ*G*_binding_ via FireDock (*57*). FireDock scoring algorithms account for van der Waals interactions, atomic contact energy, electrostatic, and additional binding free energy estimations between the target and ligand.

The advanced options for Desmond MD jobs were left as default and are as follows: Integration – RESPA integrator time step: bonded = 2.0 fs, near = 2.00, far = 6.00; Ensemble – thermostat method: Nose-Hoover chain, relaxation time = 1.0 ps; barostat method: Martyna-Tobias Klein, relaxation time = 2.0 ps, coupling style – isotopic; Coulombic interaction – short range method = cutoff, cutoff radius = 9.0 Å. The relaxation process for the NPT ensemble was left as default. Details of the relaxation process are available in the Desmond user manual: (i) minimize with solute restrained; (ii) minimize without restraints; (iii) simulate in the NVT ensemble using a Berendsen thermostat with a simulation time of 12 ps, temperature of 10°K, a fast temperature relaxation constant, velocity resampling every 1 ps, nonhydrogen solute atoms restrained; (iv) simulate in the NPT ensemble using a Berendsen thermostat and Berendsen barostat with a simulation time of 12 ps, temperature of 10 K and a pressure of 1 atm, a fast temperature relaxation constant, a slow pressure relaxation constant, velocity resampling every 1 ps, nonhydrogen solute atoms restrained; (v) simulate in the NPT ensemble using a Berendsen thermostat and Berendsen barostat with a simulation time of 24 ps, temperature of 300 K and a pressure of 1 atm, a fast temperature relaxation constant, a slow pressure relaxation constant, velocity resampling every 1 ps, nonhydrogen solute atoms restrained; (vi) simulate in the NPT ensemble using a Berendsen thermostat and Berendsen barostat with a simulation time of 24 ps, temperature of 300°K and a pressure of 1 atm, a fast temperature relaxation constant, and a normal pressure relaxation constant.

### Imaging

For TEM, nanoplastic particles and α-synuclein proteins were applied to glow-discharged 300-mesh, carbon-only, copper grids (Ladd, catalog no. 10873-25), followed by negative staining with 2% uranyl acetate (Polysciences, catalog no. 21447-25). Grids were imaged with a JEM-2100Plus Electron Microscope (JEOL, USA, Peabody, MA) operated at 120 kV with nominal magnification at ×10,000 or ×30,000. Images were collected on an AMTNanoSprint16 digital camera (AMT Imaging, Woburn, MA).

For confocal imaging, a Zeiss 880 inverted confocal microscope was used in Airyscan mode. The *x*,*y* position was set with a Märzhäuser linearly encoded stage attached to a Zeiss Observer Z.1 inverted stand. Imaging was performed with the 63× 1.4–numerical aperture (NA) oil-immersion Plan-Apochromat objective. All signals were collected with the Airyscan spatial detector with the Master gain set to 835 (488/green and 561/red) or 831 (far red), with emission was controlled by filters and beam splitters to achieve the following wavelength ranges: 488/green 495 to 550 nm, 561/red 570 to 615 nm, and 633/far red Longpass (LP) 660 nm. Images were acquired sequentially by line scanning at 1.21 μs per pixel with a line averaging of 2 and a pixel size of 0.043 μm and frame size of 1712 pixels in *x*, *y* with a zoom of 1.8. The pinhole used in confocal imaging is effectively different in Airyscan mode in which the Airyscan spatial detector collects 1.25 Airy units and Airyscan processing gives approximately 400-nm resolution in the *z* dimension. Zen black software used for image processing shows 0.4-μm section thickness for both the 488 and 561 channels and 0.5 μm for the far red channel. For three-dimensional (3D) images, *z*-stacks from optimal thicknesses underwent maximum intensity projection and were normalized using consistent intensity thresholds.

For live-cell imaging and wide-field microscopy, imaging was accomplished on a Keyence Bz-x700 microscope with a 20× Plan-Apochromat objective. Images were collected in auto-generated grids generated by the manufacturer’s controller software (Keyence Inc.) spread across wells of multiwell plastic tissue culture dishes in a semiautomated manner. All wide-field microscopy images were analyzed with ImageJ software by raters naive to experimental group identity.

For light sheet microscopy, mice were transcardially perfused with PBS followed by 4% paraformaldehyde (PFA). Whole heads were then collected and postfixed in 4% PFA overnight at 4°C. Decalcification was done by immersing the tissue in 10% EDTA for 24 hours at 4°C. The iDISCO protocol was then carried out as described (*58*, *59*). Briefly, whole heads were dehydrated in increasing methanol/H_2_O series (20, 40, 60, 80, 100, and 100%, 1 hour each), delipidated with methanol/dichloromethane (33%/66% for 3 hours) followed by pure dichloromethane (2 × 15 min), and optically cleared with ethyl cinnamate (ECI). Cleared samples were imaged on a LaVision UltraMicroscope Blaze light sheet microscope (Miltenyi Biotec) using a 4× objective (0.1 NA) equipped with an organic solvent–compatible immersion-corrected dipping cap. The excitation wavelengths were 488 and 640 nm, and emission filters used were 525/50 nm and 680/30 nm. Whole heads were imaged immersed in ECI in the horizontal orientation at a z-step size of 2 μm with InspectorPro7.5.3 software using both left- and right-side illumination and single field of view. 3D renditions were then created with Arivis Vision 4D 3.1 (Arivis AG).

### Primary neuron cultures

All procedures involving mice were approved by the Duke Institutional Animal Care and Use Committee. Hippocampi of 1-day postnatal CD1 mice pups (Charles River Laboratories, strain 022) were collected and digested using papain (20 U/ml) (Worthington, catalog no. LS003126) in Hanks’ balanced salt solution (Thermo Fisher Scientific, catalog no. 14025126) for 30 min at 37°C. The cell suspension was passed through a cell strainer with 40-μm mesh size (Fisher Scientific, catalog no. 08-771-1) before plating (Thermo Fisher Scientific, catalog no. 142485) in wells coated with poly-d-lysine (0.1 mg/ml; Thermo Fisher Scientific, catalog no. A3890401) in medium containing neurobasal (Thermo Fisher Scientific, catalog no. 21103049) supplemented with 5% fetal bovine serum, 1×B27 supplement (Thermo Fisher Scientific, catalog no. 17504044), 0.5 mM l-glutamine (Thermo Fisher Scientific, catalog no. 35050061), and penicillin-streptomycin (100 U/ml) (Thermo Fisher Scientific, catalog no. 10378016). Twelve hours later, the plating medium was removed and replaced with maintenance medium (neurobasal medium supplemented with B27 and 0.5 mM l-glutamine). The cells were cultured 7 days (DIV 7) before use in experiments.

### Endocytosis and intraneuronal localization assays

For endocytosis assays, neurons were treated with 0.001% dimethyl sulfoxide (DMSO; vehicle control), 50 μM ethyl-isopropyl amiloride, 50 μM Dynasore, 0.2 μM wortmannin, 15 μM Pitstop 2 (Abcam, catalog no. ab120687), or 2 mM methyl-β-cyclodextrin for 30 min before incubation with pHrodo-conjugated polystyrene nanoparticles or dextran particles. The average uptake level per cell was calculated by dividing fluorescence intensity by the Hoechst-positive particle (intact cell) count. For measurements related to endolysosome acidification, neurons were incubated with LysoSensor Green DND-189 (Thermo Fisher Scientific, catalog no. L7535) and then Hoechst dye (100 ng/ml), followed by washing and subsequent imaging. Relative LysoSensor green signal was calculated by dividing the fluorescence intensity by the Hoechst particle count and normalization to controls.

For analysis related to measurements of neuronal toxicity, some cultures were incubated with 2.5 μM propidium iodide (Sigma-Aldrich) and Hoechst dye (100 ng/ml) for 10 min before fixation with 4% PFA (Sigma-Aldrich). Fixed cells were stained with rabbit-NeuN (1:2000; Thermo Fisher Scientific, catalog no. 14H6L24), anti-Lamp1 (1:2000; Santa Cruz Biotechnology, catalog no. sc-19992), and anti-tau (1:1000; Thermo Fisher Scientific, catalog no. MA5-12808), with donkey anti-rabbit Alexa Fluor 555 (1:2000; Thermo Fisher Scientific, catalog no. A-31572) and donkey anti-mouse Alexa Fluor 488 (1:2000, Thermo Fisher Scientific, catalog no. A-21202) secondary antibodies, as indicated. The number of NeuN-positive cells and tau-positive pixels as a function of area was quantified using ImageJ.

For subcellular localization of internalized nanoparticles, neurons were treated with FITC-conjugated polystyrene nanoparticles (table S1) for the indicated time and cells were washed three times with PBS, fixed with 4% PFA, and immunostained for the indicated markers. The percentage of FITC pixels inside of LAMP1-positive endolysosomes in Airyscan images was quantified using ImageJ.

### Fibril-seeded α-synuclein aggregation in primary neurons

Neurons were treated with the indicated concentration of preformed sonicated α-synuclein fibrils (fig. S1). At end points, cells were then washed with PBS, fixed with 4% PFA, and immunostained with anti-tau (1:1000; Invitrogen, MA5-12808) and anti–pS129–α-synuclein (1:2000; Abcam, ab51253) followed by staining with donkey anti-rabbit Alexa Fluor 555 (1:2000: Thermo Fisher Scientific, catalog no. A-31572) and donkey anti-mouse Alexa Fluor 488 (1:2000; Thermo Fisher Scientific, catalog no. A-21202), with 4′,6-diamidino-2-phenylindole (DAPI) (Sigma-Aldrich) dye. In obtained images, the area of pS129–α-synuclein–positive pixels was divided by the area of tau-positive pixels. To analyze the soluble and insoluble development of α-synuclein in primary hippocampal neurons, cells were lysed in 25 mM tris (pH 7.4), 100 mM NaCl, 1× protease inhibitors (Roche, catalog no. 04693116001), and 1× phosphatase inhibitors (Roche, catalog no. 4906845001), followed by centrifugation at 50,000*g*. The supernatant was collected as the soluble fraction. The pellets were resuspended in a lysis buffer with 2× Laemmli buffer and analyzed by SDS-PAGE followed by immunoblotting using anti–pS129–α-synuclein (1:5000; Abcam, catalog no. ab51253), anti–α-synuclein (1:5000; Abcam, catalog no. ab1903), or anti–human α-synuclein (1:5000; Abcam, catalog no. ab80627) antibodies.

### Mouse intracranial injections and tissue staining

Intracranial injections of nanoparticles and/or α-synuclein fibrils, or vehicle control were performed as previously described (*44*). Male wild-type outbred CD1 mice obtained from Charles River Laboratories were randomized to groups spread across multiple cages. Mice were used for experiments at 3 months of age. While power calculations were not performed, because the measures and models reported here use a model not previously described, group sizes of *N* = 8 to 12 were approximated on the basis of a previous publication that evaluated pS129–α-synuclein pathology resultant from the injection of human preformed α-synuclein fibrils into the dorsal striatum in wild-type mice (*45*). Mice were anesthetized with vaporized isoflurane and dexdomitor on a stereotaxic frame (Kopf Instruments). Solutions for injection were drawn to a 32-gauge custom needle (Hamilton) fitted to a gas-tight syringe and controlled by a digital pump (Harvard Apparatus). Three microliters of solution containing the indicated amount of plastic or α-synuclein fibrils, or vehicle control, was injected into the right dorsal striatum relative to bregma: 1.0 mm anterior, 1.85 mm lateral, and 3.0 mm ventral relative to the skull. Scalp incisions were closed by suture. Three days or 2 months after injection (as indicated), mice were deeply anesthetized with isoflurane and transcardially perfused with PBS followed by freshly prepared 4% PFA buffered in PBS. Brains were removed, postfixed for 24 hours in 4% PFA and PBS solution, floated into 30% sucrose PBS solution for 2 days, and then cut to 40 μm on a freezing microtome (Leica SM2010 R Sliding Microtome).

Sections were incubated in an antigen retrieval buffer [10 mM sodium citrate and 0.05% Tween 20 (pH 6.0)] for 1 hour with gentle rocking at 37°C before being rinsed and incubated with tris-buffered saline (TBS; Thermo Fisher Scientific, catalog no. J60764.K2) with 5% donkey serum and 0.3% Triton X-100 for 1 hour. To detect α-synuclein pathology, sections were incubated with primary antibodies including anti–tyrosine hydroxylase (1:2000; MilliporeSigma, catalog no. AB1542), anti–α-synuclein (pS129) antibody (1:4000; catalog no. EP1536Y, Abcam), or anti-NeuN antibody (1:2000; Thermo Fisher Scientific, catalog no. 14H6L24) in TBS buffer supplemented with 5% donkey serum and 0.1% Triton X-100 for 24 hours before incubations with secondary antibodies including donkey anti-sheep Alexa Fluor 555 (1:2000; Thermo Fisher Scientific, catalog no. A-21436), donkey anti-rabbit Alexa Fluor 647 (1:2000; Thermo Fisher Scientific, catalog no. A32795), or donkey anti-rabbit Alexa Fluor 555 (1:2000; Thermo Fisher Scientific, catalog no. A-31572) for 24 hours. Sections were mounted on superfrost slides with ProLong Gold Antifade Mountant (Thermo Fisher Scientific, P36930). The number of pS129–α-synuclein–positive cells and percent of area occupied by pS129–α-synuclein were calculated using ImageJ.

### Statistical analyses

Analyses were performed by investigators blinded to sample identity until final data curation using coded identifiers for sample lysates, slides, and mice. GraphPad Prism 9 or IBM SPSS Statistics was used in all statistical analyses. Data distributions were assessed by Shapiro-Wilk tests. Data with normal distribution were assessed by one-way analysis of variance (ANOVA) (with Tukey’s group mean comparison post hoc test) or a two-tailed *t* test for group comparisons. Data with nonnormal distributions were assessed with Mann-Whitney tests. *P* values of <0.05 were considered significant. Datasets were plotted in GraphPad Prism or in RStudio.

## References

[1] M. Carbery, W. O’Connor, T. Palanisami, Trophic transfer of microplastics and mixed contaminants in the marine food web and implications for human health. Environ. Int. 115, 400–409 (2018). 29653694 10.1016/j.envint.2018.03.007

[2] M. Enfrin, L. F. Dumée, J. Lee, Nano/microplastics in water and wastewater treatment processes—Origin, impact and potential solutions. Water Res. 161, 621–638 (2019). 31254888 10.1016/j.watres.2019.06.049

[3] A. A. Koelmans, N. H. Mohamed Nor, E. Hermsen, M. Kooi, S. M. Mintenig, J. De France, Microplastics in freshwaters and drinking water: Critical review and assessment of data quality. Water Res. 155, 410–422 (2019). 30861380 10.1016/j.watres.2019.02.054 PMC6449537

[4] R. E. J. Schnurr, V. Alboiu, M. Chaudhary, R. A. Corbett, M. E. Quanz, K. Sankar, H. S. Srain, V. Thavarajah, D. Xanthos, T. R. Walker, Reducing marine pollution from single-use plastics (SUPs): A review. Mar. Pollut. Bull. 137, 157–171 (2018). 30503422 10.1016/j.marpolbul.2018.10.001

[5] A. E. Schwarz, T. N. Ligthart, E. Boukris, T. van Harmelen, Sources, transport, and accumulation of different types of plastic litter in aquatic environments: A review study. Mar. Pollut. Bull. 143, 92–100 (2019). 31789171 10.1016/j.marpolbul.2019.04.029

[6] K. N. Fotopoulou, H. K. Karapanagioti, Surface properties of beached plastic pellets. Mar. Environ. Res. 81, 70–77 (2012). 23013745 10.1016/j.marenvres.2012.08.010

[7] W. Wei, Y. Li, M. Lee, N. Andrikopoulos, S. Lin, C. Chen, D. T. Leong, F. Ding, Y. Song, P. C. Ke, Anionic nanoplastic exposure induces endothelial leakiness. Nat. Commun. 13, 4757 (2022). 35963861 10.1038/s41467-022-32532-5 PMC9376074

[8] H. A. Leslie, M. J. M. van Velzen, S. H. Brandsma, A. D. Vethaak, J. J. Garcia-Vallejo, M. H. Lamoree, Discovery and quantification of plastic particle pollution in human blood. Environ. Int. 163, 107199 (2022). 35367073 10.1016/j.envint.2022.107199

[9] L. Hoelting, B. Scheinhardt, O. Bondarenko, S. Schildknecht, M. Kapitza, V. Tanavde, B. Tan, Q. Y. Lee, S. Mecking, M. Leist, S. Kadereit, A 3-dimensional human embryonic stem cell (hESC)-derived model to detect developmental neurotoxicity of nanoparticles. Arch. Toxicol. 87, 721–733 (2013). 23203475 10.1007/s00204-012-0984-2 PMC3604581

[10] S. A. Kulkarni, S.-S. Feng, Effects of particle size and surface modification on cellular uptake and biodistribution of polymeric nanoparticles for drug delivery. Pharm. Res. 30, 2512–2522 (2013). 23314933 10.1007/s11095-012-0958-3

[11] S. Kashiwada, Distribution of nanoparticles in the see-through medaka (Oryzias latipes). Environ. Health Perspect. 114, 1697–1702 (2006). 17107855 10.1289/ehp.9209 PMC1665399

[12] X. Liu, Y. Zhao, J. Dou, Q. Hou, J. Cheng, X. Jiang, Bioeffects of inhaled nanoplastics on neurons and alteration of animal behaviors through deposition in the brain. Nano Lett. 22, 1091–1099 (2022). 35089039 10.1021/acs.nanolett.1c04184

[13] C.-S. Yang, C.-H. Chang, P.-J. Tsai, W.-Y. Chen, F.-G. Tseng, L.-W. Lo, Nanoparticle-based in vivo investigation on blood-brain barrier permeability following ischemia and reperfusion. Anal. Chem. 76, 4465–4471 (2004). 15283589 10.1021/ac035491v

[14] L. C. Katz, A. Burkhalter, W. J. Dreyer, Fluorescent latex microspheres as a retrograde neuronal marker for in vivo and in vitro studies of visual cortex. Nature 310, 498–500 (1984). 6205278 10.1038/310498a0

[15] H. Qian, X. Kang, J. Hu, D. Zhang, Z. Liang, F. Meng, X. Zhang, Y. Xue, R. Maimon, S. F. Dowdy, N. K. Devaraj, Z. Zhou, W. C. Mobley, D. W. Cleveland, X.-D. Fu, Reversing a model of Parkinson’s disease with in situ converted nigral neurons. Nature 582, 550–556 (2020). 32581380 10.1038/s41586-020-2388-4 PMC7521455

[16] K. Murali, K. Kenesei, Y. Li, K. Demeter, Z. Környei, E. Madarász, Uptake and bio-reactivity of polystyrene nanoparticles is affected by surface modifications, ageing and LPS adsorption: In vitro studies on neural tissue cells. Nanoscale 7, 4199–4210 (2015). 25673096 10.1039/c4nr06849a

[17] A. Lesniak, D. Kilinc, A. Blasiak, G. Galea, J. C. Simpson, G. U. Lee, Rapid growth cone uptake and dynein-mediated axonal retrograde transport of negatively charged nanoparticles in neurons is dependent on size and cell type. Small 15, e1803758 (2019). 30565853 10.1002/smll.201803758

[18] GBD 2015 Neurological Disorders Collaborator Group, Global, regional, and national burden of neurological disorders during 1990-2015: A systematic analysis for the Global Burden of Disease Study 2015. Lancet Neurol. 16, 877–897 (2017). 28931491 10.1016/S1474-4422(17)30299-5 PMC5641502

[19] E. R. Dorsey, T. Sherer, M. S. Okun, B. R. Bloem, The emerging evidence of the Parkinson pandemic. J. Parkinsons Dis. 8, S3–S8 (2018). 30584159 10.3233/JPD-181474 PMC6311367

[20] D. Erskine, D. Koss, V. I. Korolchuk, T. F. Outeiro, J. Attems, I. McKeith, Lipids, lysosomes and mitochondria: Insights into Lewy body formation from rare monogenic disorders. Acta Neuropathol. 141, 511–526 (2021). 33515275 10.1007/s00401-021-02266-7 PMC7952289

[21] H. Mohammad-Beigi, M. Zanganeh, C. Scavenius, H. Eskandari, A. Farzadfard, S. A. Shojaosadati, J. J. Enghild, D. E. Otzen, A. K. Buell, D. S. Sutherland, A protein corona modulates interactions of α-synuclein with nanoparticles and alters the rates of the microscopic steps of amyloid formation. ACS Nano 16, 1102–1118 (2022). 34982538 10.1021/acsnano.1c08825

[22] D. Kim, J. M. Yoo, H. Hwang, J. Lee, S. H. Lee, S. P. Yun, M. J. Park, M. Lee, S. Choi, S. H. Kwon, S. Lee, S.-H. Kwon, S. Kim, Y. J. Park, M. Kinoshita, Y.-H. Lee, S. Shin, S. R. Paik, S. J. Lee, S. Lee, B. H. Hong, H. S. Ko, Graphene quantum dots prevent α-synucleinopathy in Parkinson’s disease. Nat. Nanotechnol. 13, 812–818 (2018). 29988049 10.1038/s41565-018-0179-y PMC6351226

[23] X. Yuan, Y. Yang, D. Xia, L. Meng, M. He, C. Liu, Z. Zhang, Silica nanoparticles promote α-synuclein aggregation and Parkinson’s disease pathology. Front. Neurosci. 15, 807988 (2021). 35095403 10.3389/fnins.2021.807988 PMC8792744

[24] A. Sokratian, J. Ziaee, K. Kelly, A. Chang, N. Bryant, S. Wang, E. Xu, J. Y. Li, S.-H. Wang, J. Ervin, S. M. Swain, R. A. Liddle, A. B. West, Heterogeneity in α-synuclein fibril activity correlates to disease phenotypes in Lewy body dementia. Acta Neuropathol. 141, 547–564 (2021). 33641009 10.1007/s00401-021-02288-1 PMC8055043

[25] M. E. Bernis, J. T. Babila, S. Breid, K. A. Wüsten, U. Wüllner, G. Tamgüney, Prion-like propagation of human brain-derived α-synuclein in transgenic mice expressing human wild-type α-synuclein. Acta Neuropathol. Commun. 3, 75 (2015). 26612754 10.1186/s40478-015-0254-7 PMC4660655

[26] B. G. Wilhelm, S. Mandad, S. Truckenbrodt, K. Kröhnert, C. Schäfer, B. Rammner, S. J. Koo, G. A. Claßen, M. Krauss, V. Haucke, H. Urlaub, S. O. Rizzoli, Composition of isolated synaptic boutons reveals the amounts of vesicle trafficking proteins. Science 344, 1023–1028 (2014). 24876496 10.1126/science.1252884

[27] N. Bossa, J. M. Sipe, W. Berger, K. Scott, A. Kennedy, T. Thomas, C. O. Hendren, M. R. Wiesner, Quantifying mechanical abrasion of MWCNT nanocomposites used in 3D printing: Influence of CNT content on abrasion products and rate of microplastic production. Environ. Sci. Technol. 55, 10332–10342 (2021). 34264058 10.1021/acs.est.0c02015 PMC10084403

[28] J. M. Sipe, N. Bossa, W. Berger, N. von Windheim, K. Gall, M. R. Wiesner, From bottle to microplastics: Can we estimate how our plastic products are breaking down? Sci. Total Environ. 814, 152460 (2022). 34973311 10.1016/j.scitotenv.2021.152460

[29] S. Kim, S.-H. Kwon, T.-I. Kam, N. Panicker, S. S. Karuppagounder, S. Lee, J. H. Lee, W. R. Kim, M. Kook, C. A. Foss, C. Shen, H. Lee, S. Kulkarni, P. J. Pasricha, G. Lee, M. G. Pomper, V. L. Dawson, T. M. Dawson, H. S. Ko, Transneuronal propagation of pathologic α-synuclein from the gut to the brain models Parkinson’s disease. Neuron 103, 627–641.e7 (2019). 31255487 10.1016/j.neuron.2019.05.035 PMC6706297

[30] S. Kihara, S. Ghosh, D. R. McDougall, A. E. Whitten, J. P. Mata, I. Köper, D. J. McGillivray, Structure of soft and hard protein corona around polystyrene nanoplastics—Particle size and protein types. Biointerphases 15, 051002 (2020). 32948094 10.1116/6.0000404

[31] S. Kihara, N. J. van der Heijden, C. K. Seal, J. P. Mata, A. E. Whitten, I. Köper, D. J. McGillivray, Soft and hard interactions between polystyrene nanoplastics and human serum albumin protein corona. Bioconjug. Chem. 30, 1067–1076 (2019). 30821961 10.1021/acs.bioconjchem.9b00015

[32] E. Rhoades, T. F. Ramlall, W. W. Webb, D. Eliezer, Quantification of alpha-synuclein binding to lipid vesicles using fluorescence correlation spectroscopy. Biophys. J. 90, 4692–4700 (2006). 16581836 10.1529/biophysj.105.079251 PMC1471845

[33] T. Dou, L. Zhou, D. Kurouski, Unravelling the structural organization of individual α-synuclein oligomers grown in the presence of phospholipids. J. Phys. Chem. Lett. 12, 4407–4414 (2021). 33945282 10.1021/acs.jpclett.1c00820

[34] M. Konno, T. Hasegawa, T. Baba, E. Miura, N. Sugeno, A. Kikuchi, F. C. Fiesel, T. Sasaki, M. Aoki, Y. Itoyama, A. Takeda, Suppression of dynamin GTPase decreases α-synuclein uptake by neuronal and oligodendroglial cells: A potent therapeutic target for synucleinopathy. Mol. Neurodegener. 7, 38 (2012). 22892036 10.1186/1750-1326-7-38 PMC3479026

[35] E. Macia, M. Ehrlich, R. Massol, E. Boucrot, C. Brunner, T. Kirchhausen, Dynasore, a cell-permeable inhibitor of dynamin. Dev. Cell 10, 839–850 (2006). 16740485 10.1016/j.devcel.2006.04.002

[36] L. von Kleist, W. Stahlschmidt, H. Bulut, K. Gromova, D. Puchkov, M. J. Robertson, K. A. MacGregor, N. Tomilin, A. Pechstein, N. Chau, M. Chircop, J. Sakoff, J. P. von Kries, W. Saenger, H.-G. Kräusslich, O. Shupliakov, P. J. Robinson, A. McCluskey, V. Haucke, Role of the clathrin terminal domain in regulating coated pit dynamics revealed by small molecule inhibition. Cell 146, 471–484 (2011). 21816279 10.1016/j.cell.2011.06.025

[37] Y. W. H. Koh, Y. Hung, N. Tuladhar, Z. Xiao, D. L. Brown, N. D. Condon, J. L. Stow, Live fluorescence, inverse imaging of cell ruffling, and macropinocytosis. J. Vis. Exp. 5, (2021). 10.3791/62870 34424243

[38] Y. Ye, L. Hui, K. L. Lakpa, Y. Xing, H. Wollenzien, X. Chen, J. X. Zhao, J. D. Geiger, Effects of silica nanoparticles on endolysosome function in primary cultured neurons. Can. J. Physiol. Pharmacol. 97, 297–305 (2019). 30312546 10.1139/cjpp-2018-0401 PMC6443500

[39] A. N. Sacino, M. M. Brooks, P. Chakrabarty, K. Saha, H. Khoshbouei, T. E. Golde, B. I. Giasson, Proteolysis of α-synuclein fibrils in the lysosomal pathway limits induction of inclusion pathology. J. Neurochem. 140, 662–678 (2017). 27424880 10.1111/jnc.13743 PMC5452425

[40] S. K. Mak, A. L. McCormack, A. B. Manning-Bog, A. M. Cuervo, D. A. Di Monte, Lysosomal degradation of α-synuclein in vivo. J. Biol. Chem. 285, 13621–13629 (2010). 20200163 10.1074/jbc.M109.074617 PMC2859524

[41] A. M. Cuervo, L. Stefanis, R. Fredenburg, P. T. Lansbury, D. Sulzer, Impaired degradation of mutant α-synuclein by chaperone-mediated autophagy. Science 305, 1292–1295 (2004). 15333840 10.1126/science.1101738

[42] J. L. Webb, B. Ravikumar, J. Atkins, J. N. Skepper, D. C. Rubinsztein, α-Synuclein is degraded by both autophagy and the proteasome. J. Biol. Chem. 278, 25009–25013 (2003). 12719433 10.1074/jbc.M300227200

[43] M. Neumann, P. J. Kahle, B. I. Giasson, L. Ozmen, E. Borroni, W. Spooren, V. Müller, S. Odoy, H. Fujiwara, M. Hasegawa, T. Iwatsubo, J. Q. Trojanowski, H. A. Kretzschmar, C. Haass, Misfolded proteinase K-resistant hyperphosphorylated alpha-synuclein in aged transgenic mice with locomotor deterioration and in human α-synucleinopathies. J. Clin. Invest. 110, 1429–1439 (2002). 12438441 10.1172/JCI15777 PMC151810

[44] H. Abdelmotilib, T. Maltbie, V. Delic, Z. Liu, X. Hu, K. B. Fraser, M. S. Moehle, L. Stoyka, N. Anabtawi, V. Krendelchtchikova, L. A. Volpicelli-Daley, A. West, α-Synuclein fibril-induced inclusion spread in rats and mice correlates with dopaminergic neurodegeneration. Neurobiol. Dis. 105, 84–98 (2017). 28576704 10.1016/j.nbd.2017.05.014 PMC5701756

[45] K. C. Luk, D. J. Covell, V. M. Kehm, B. Zhang, I. Y. Song, M. D. Byrne, R. M. Pitkin, S. C. Decker, J. Q. Trojanowski, V. M.-Y. Lee, Molecular and biological compatibility with host α-synuclein influences fibril pathogenicity. Cell Rep. 16, 3373–3387 (2016). 27653697 10.1016/j.celrep.2016.08.053 PMC5087609

[46] L. C. Katz, D. M. Iarovici, Green fluorescent latex microspheres: A new retrograde tracer. Neuroscience 34, 511–520 (1990). 2333155 10.1016/0306-4522(90)90159-2

[47] K. C. Luk, V. Kehm, J. Carroll, B. Zhang, P. O’Brien, J. Q. Trojanowski, V. M.-Y. Lee, Pathological α-synuclein transmission initiates Parkinson-like neurodegeneration in nontransgenic mice. Science 338, 949–953 (2012). 23161999 10.1126/science.1227157 PMC3552321

[48] S. Shan, Y. Zhang, H. Zhao, T. Zeng, X. Zhao, Polystyrene nanoplastics penetrate across the blood–brain barrier and induce activation of microglia in the brain of mice. Chemosphere 298, 134261 (2022). 35302003 10.1016/j.chemosphere.2022.134261

[49] D. M. Mitrano, P. Wick, B. Nowack, Placing nanoplastics in the context of global plastic pollution. Nat. Nanotechnol. 16, 491–500 (2021). 33927363 10.1038/s41565-021-00888-2

[50] W. Fu, J. Min, W. Jiang, Y. Li, W. Zhang, Separation, characterization and identification of microplastics and nanoplastics in the environment. Sci. Total Environ. 721, 137561 (2020). 32172100 10.1016/j.scitotenv.2020.137561

[51] V. Kopatz, K. Wen, T. Kovács, A. S. Keimowitz, V. Pichler, J. Widder, A. D. Vethaak, O. Hollóczki, L. Kenner, Micro- and nanoplastics breach the blood–brain barrier (BBB): Biomolecular corona’s role revealed. Nanomaterials 13, 1404 (2023). 37110989 10.3390/nano13081404 PMC10141840

[52] B. R. De Miranda, S. M. Goldman, G. W. Miller, J. T. Greenamyre, E. R. Dorsey, Preventing Parkinson’s disease: An environmental agenda. J. Parkinsons Dis. 12, 45–68 (2022). 34719434 10.3233/JPD-212922 PMC8842749

[53] R. L. Wallings, S. W. Humble, M. E. Ward, R. Wade-Martins, Lysosomal dysfunction at the centre of Parkinson’s disease and frontotemporal dementia/amyotrophic lateral sclerosis. Trends Neurosci. 42, 899–912 (2019). 31704179 10.1016/j.tins.2019.10.002 PMC6931156

[54] C. H. Adler, D. J. Connor, J. G. Hentz, M. N. Sabbagh, J. N. Caviness, H. A. Shill, B. Noble, T. G. Beach, Incidental Lewy body disease: Clinical comparison to a control cohort. Mov. Disord. 25, 642–646 (2010). 20175211 10.1002/mds.22971 PMC2935627

[55] A. Mehdiani, A. Maier, A. Pinto, M. Barth, P. Akhyari, A. Lichtenberg, An innovative method for exosome quantification and size measurement. J. Vis. Exp. 50974, (2015). 10.3791/50974 PMC4354536 25650897

[56] M. J. Abraham, T. Murtola, R. Schulz, S. Páll, J. C. Smith, B. Hess, E. Lindahl, GROMACS: High performance molecular simulations through multi-level parallelism from laptops to supercomputers. SoftwareX 1-2, 19–25 (2015).

[57] N. Andrusier, R. Nussinov, H. J. Wolfson, FireDock: Fast interaction refinement in molecular docking. Proteins 69, 139–159 (2007). 17598144 10.1002/prot.21495

[58] N. Renier, E. L. Adams, C. Kirst, Z. Wu, R. Azevedo, J. Kohl, A. E. Autry, L. Kadiri, K. Umadevi Venkataraju, Y. Zhou, V. X. Wang, C. Y. Tang, O. Olsen, C. Dulac, P. Osten, M. Tessier-Lavigne, Mapping of brain activity by automated volume analysis of immediate early genes. Cell 165, 1789–1802 (2016). 27238021 10.1016/j.cell.2016.05.007 PMC4912438

[59] A. Ertürk, F. Bradke, High-resolution imaging of entire organs by 3-dimensional imaging of solvent cleared organs (3DISCO). Exp. Neurol. 242, 57–64 (2013). 23124097 10.1016/j.expneurol.2012.10.018

